# On Prediction of a Novel Chiral Material Y_2_H_3_O(OH): A Hydroxyhydride Holding Hydridic and Protonic Hydrogens

**DOI:** 10.3390/ma13040994

**Published:** 2020-02-22

**Authors:** Aleksandr Pishtshev, Evgenii Strugovshchikov, Smagul Karazhanov

**Affiliations:** 1 Institute of Physics, University of Tartu, W.Ostwaldi 1, 50411 Tartu, Estonia; aleksandr.pishtshev@ut.ee; 2 Department for Solar Energy, Institute for Energy Technology, 2007 Kjeller, Norway; smagul.karazhanov@ife.no

**Keywords:** chiral materials, oxyhydride, mixed-anion chemistry, crystal structure prediction

## Abstract

Examination of possible pathways of how oxygen atoms can be added to a yttrium oxyhydride system allowed us to predict new derivatives such as hydroxyhydrides possessing the composition M_2_H_3_O(OH) (M = Y, Sc, La, and Gd) in which three different anions (H^-^, O^2^−, and OH^-^) share the common chemical space. The crystal data of the solid hydroxyhydrides obtained on the base of DFT modeling correspond to the tetragonal structure that is characterized by the chiral space group $P4_{1}$. The analysis of bonding situation in M_2_H_3_O(OH) showed that the microscopic mechanism governing chemical transformations is caused by the displacements of protons which are induced by interaction with oxygen atoms incorporated into the crystal lattice of the bulk oxyhydride. The oxygen-mediated transformation causes a change in the charge state of some adjacent hydridic sites, thus forming protonic sites associated with hydroxyl groups. The predicted materials demonstrate a specific charge ordering that is associated with the chiral structural organization of the metal cations and the anions because their lattice positions form helical curves spreading along the tetragonal axis. Moreover, the effect of spatial twisting of the H^-^ and H^+^ sites provides additional linking via strong dihydrogen bonds. The structure–property relationships have been investigated in terms of structural, mechanical, electron, and optical features. It was shown that good polar properties of the materials make them possible prototypes for the design of nonlinear optical systems.

## 1. Introduction

Design of inorganic compounds containing different anions attracts a lot of attention because it provides an opportunity to develop new functionality of material properties [1,2,3,4,5]. Over the past decade, the anion exchange of metal oxide for a hydride ion has become the subject of intensive research in the mixed-anion chemistry [6,7,8,9,10,11]. The original synthesis routes based on topotactic solid-state reactions and high-pressure methods were reported for a number of mixed-oxyanion crystalline phases with different levels of H^-^/O^2^− exchange [12,13,14,15,16,17,18,19,20,21,22]. Recently, investigations of the controllable oxidation of a metal-hydrogen mixture [23] have led to the synthesis of transition metal and rare-earth metal oxyhydride-type materials [24,25,26,27] which have demonstrated remarkable stability at room temperatures and atmospheric pressure. Varying levels of oxidation afforded a good performance of the synthesized films with respect to photochromic properties; their promising applications in optoelectronics is presently of growing interest [24]. One of the key results of our previous work [28] on yttrium oxyhydrides is that these compounds may crystallize over a wide range of stoichiometric compositions, the most part of which can be associated with two homologous series, Y_(2n+m)/3_H_m_O_n_ and Y_(4n+2m)/5_H_m_O_n_.

The remarkable feature of the metal–hydrogen bond is that its cleavage may proceed via three reaction pathways which correspond to releasing a proton, hydrogen radical, or hydride [29]. Based on this common scheme, it could therefore be interesting to investigate the possibility of yttrium hydride oxidation routes that might embody protons and hydride anions in an amount sufficient to overcome a hydridic character of hydrogen centers. The feasibility of joint accommodation of H^-^ and H^+^ (via OH^-^ anions) in the lattice was studied by Hayashi et al. [30] for mayenite and apatite crystalline hosts. A year later, Masuda and coworkers [22] reported on a study of deuterated versions of a titanium perovskite oxyhydride in which it was experimentally shown that H^-^/H^+^ concerted coexistence can be realized at ambient conditions. In the present work, we will show that the stabilization of hydrogen confinement can be accomplished through selective partitioning of the space which the anions are sharing in the crystal lattice of a oxyhydride system. Two factors support our assertion. The first one is that, by incorporating oxygen into the host hydride system, one can create an electron-withdrawing group stronger than that represented by the hydride anion. The second factor is predetermined by the high symmetry of the host lattice, which, in turn, affords a crystalline environment with interstitials sufficient to accommodate protonic hydrogens. Our idea is that the combination of these factors may allow facile accommodations of an oxogroup in a structural framework where the oxygen may redistribute as much electron density as possible both on the metal center and the hydride anion. Of course, this quest presents a challenge in the prediction of a stable lattice structure of which the bonding patterns might precede the conventional merging H^-^ + H^+^ -> H_2_. Thus, a crystal chemical model of the yttrium oxyhydride system will be the focus of the present study. Correspondingly, the main goal is to design a stable lattice configuration that may afford the separable partitioning of the H^-^ and H^+^ species in real crystallographic space. Suggesting oxygen-poor conditions, we have conducted theoretical modeling and simulations of the oxyhydride system in terms of various structural transformations. Our initial purpose was to explore in which way an oxidative addition of oxygen into the host lattice of yttrium hydride may generate such electron-deficient species as H^+^ cations. By considering the energetics and stability of different intermediate configurations of cubic and tetragonal symmetries, we have arrived at a scenario that formally features the coupling of the cationic oxyhydridic moiety [H_3_Y_2_O]^+^ and the protic end (OH)^-^. It turned out that just a periodic lattice structure with an chiral symmetry affords the full spatial arrangement of hydridic and protonic hydrogens.

## 2. Results and Discussion

**Figure 1:**
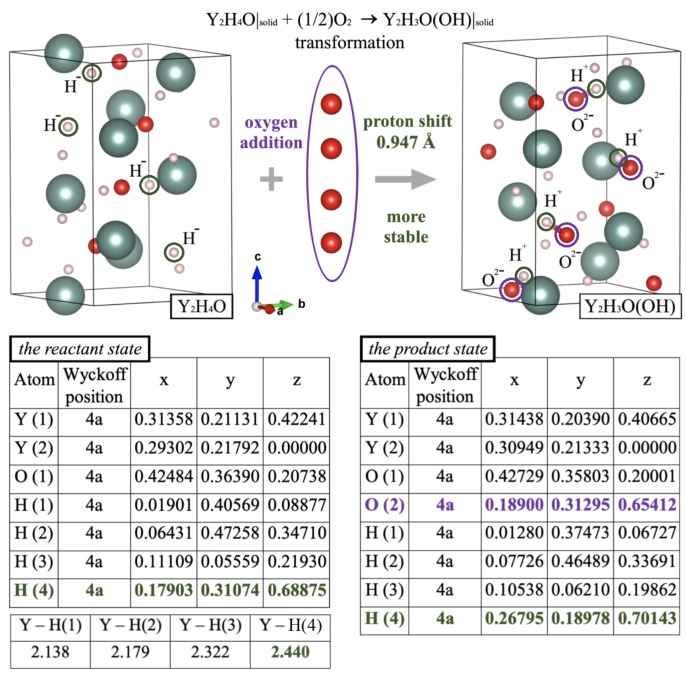
A schematic view of the rearrangement effect rendered as a comparison of unit cells related to less stable and more stable compositions. The color codes of atoms: Y—green, H—pink, and O—red spheres. Models of crystal packing are shown in terms positional characteristics. The H(4) and O(2) orbits which are active in the hydrogen migration are highlighted in green and in purple, respectively. Also, the “lost” hydridic hydrogens of the H(4) orbit (the left cell) converted to protons (the right cell) are ringed in green, while the added oxygens comprising the O(2) orbit are ringed in purple. The combination of orbits O(2) and H(4) generates four hydroxy groups in the unit cell of Y_2_H_3_O(OH).

**Figure 2:**
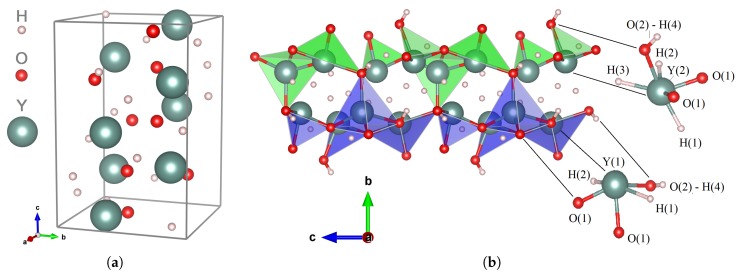
Predicted crystal structure of Y_2_H_3_O(OH): The structural parameters are given in Table 1. (**a**) A schematic view of the unit cell along the tetragonal *c* axis. (**b**) A characteristic pattern of polyhedral chains viewed along *a* axis.

**Figure 3:**
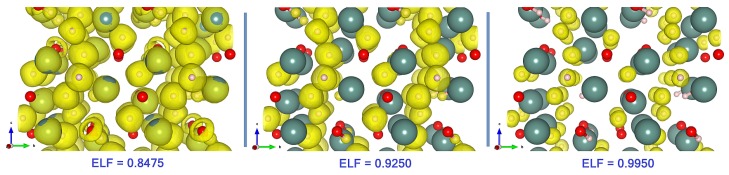
Valence electron localization function (ELF) isosurfaces evaluated for the different values of ELF: These values are given at the bottom of the visualizations. The color codes of the atoms are the same as in Figure 2. The ELF isosurface at ELF = 0.8475 contains the torus-shaped domen typical for the hydroxide anion.

**Figure 4:**
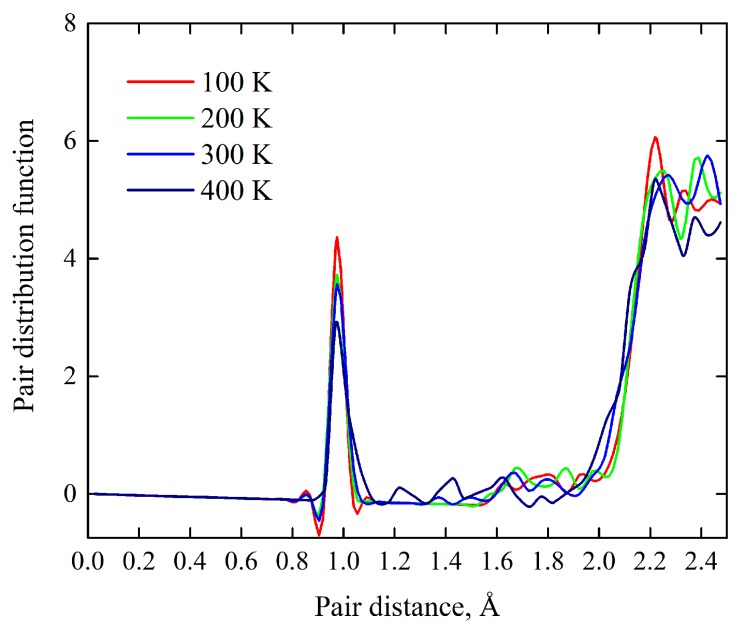
Temperature behavior of the pair distribution function for the O−H, Y−H, and Y−O bond distances as it follows from MD simulations performed over the range 0–400 K.

**Figure 5:**
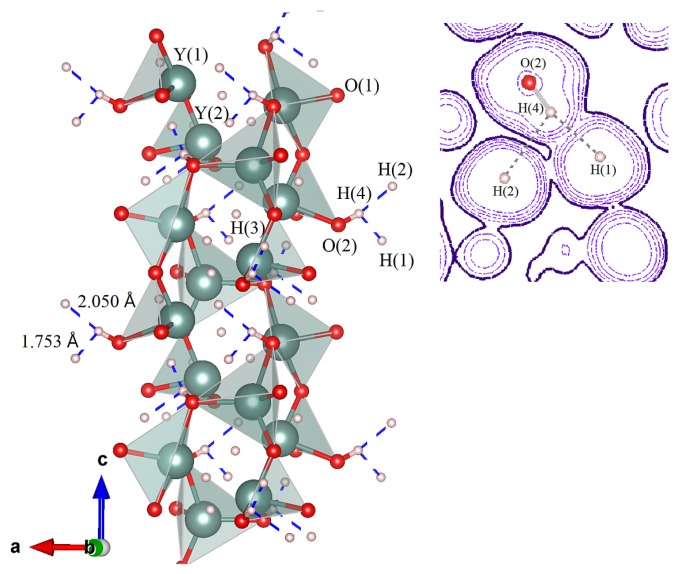
Chains of yttrium polyhedral units linked by dihydrogen bonds in the Y_2_H_3_O(OH) crystal structure: The blue dashed lines denote the relevant H···H contacts. The color codes of atoms are the same as in Figure 2. At the right, 2D contour plot of the valence electron density, calculated at ELF=0.8475, presents O(2)−H(4)···H(1) and O(2)−H(4)···H(2) dihydrogen bonding connections. According to the AIM protocol for charge densities, the minimum distances to a zero flux surface are located at distances of about 0.809, 0.816, and 0.159 Å for the H(1), H(2), and H(4) sites, respectively.

**Figure 6:**
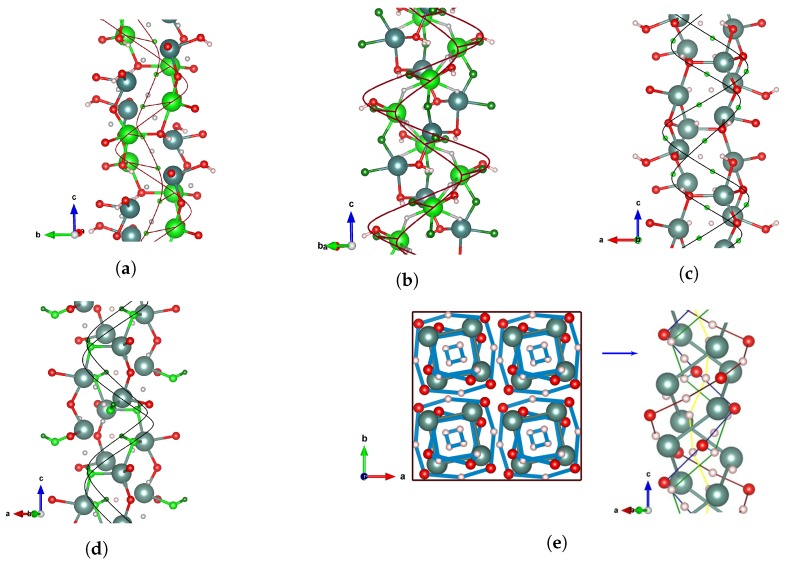
Scheme of a chiral packing composed of the circular helices with pitch of 9.264 Å: The color codes of atoms are the same as in Figure 2. The helical structures were parametrized in terms of functions x(t)=acos(t), y(t)=asin(t), and z(t)=bt, where *a* is the radius and the ratio b/a represents the slope. (**a**) Helical chains formed by Y(1) and H(3). Y(1): a=2.17 Å, b/a=0.68; H(3): a=0.75 Å, b/a=1.97. (**b**) Helical chains formed by Y(2) and O(1). Y(2): a=2.17 Å, b/a=0.68; O(1): a=3.28 Å, b/a=0.45. (**c**) Helical chains formed by H(1) and H(2). H(1): a=2.27 Å, b/a=0.65; H(2): a=2.84 Å, b/a=0.52. (**d**) Helical chains formed by O(2) and H(4). O(2): a=2.17 Å, b/a=0.68; H(4): a=1.94 Å, b/a=0.76. (**e**) Packing motif of the helical structure in the unit cell. A column composed of six joined helical chains represents the building block of the chiral framework of Y_2_H_3_O(OH). The internal tunnel is filled by the single H(3) helical chain.

**Figure 7:**
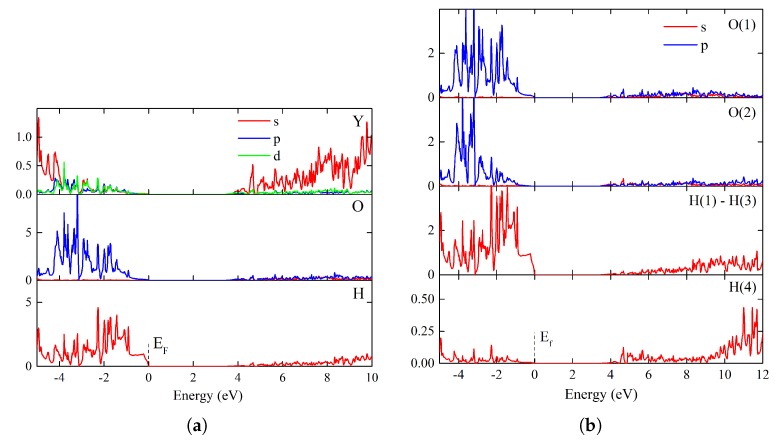
Electron structure of Y_2_H_3_O(OH) in terms of density of states (DOS):: The Fermi level is shifted to a zero value. The highest valence band state and the lowest conduction band state at the Brillouin zone center determine the fundamental band-gap width of 3.4 eV. (**a**) Element-projected partial density of states. (**b**) Site-projected partial density of states. H(1)–H(3) positions relate to the hydride anion; H(4) describes the proton in the hydroxide anion.

**Figure 8:**
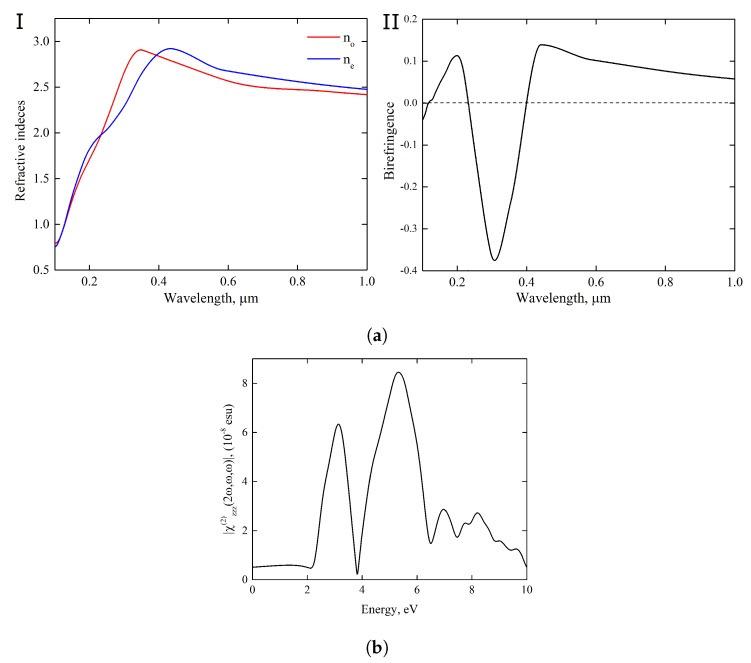
Optical responses: (**a**) The phenomena of anomalous dispersion of refractive indicies (I) and of anomalous birefringence ($\Delta n=n_{e}-n_{o}$) (II) in uniaxial Y_2_H_3_O(OH). The optic axis is fixed in the *z* direction. In subsection (II), crossing of the dashed line with the birefringence curve indicates λ= 117, 231, and 401 nm as isotropic points in the given spectral region. (**b**) Prediction of the second-harmonic generation (SHG) spectrum of Y_2_H_3_O(OH) represented in terms of the spectral behavior of the absolute value of the second-order susceptibility tensor component $\chi_{zzz}\left(2\omega ,\omega ,\omega \right)$.

The main results of the present work on the structural chemistry, chemical bonding, the energetics, and the chiral microstructure-property relations of Y_2_H_3_O(OH) are given in Table 1, Table 2, Table 3, Table 4, Table 5 and Table 6 and Figure 1, Figure 2, Figure 3, Figure 4, Figure 5, Figure 6, Figure 7 and Figure 8. The details are analyzed in subsequent subsections.

### 2.1. Modeling

A set of procedures we used in the present study to simulate the oxidation-mediated changes of crystal structures is described in the Section “Methods”. The main purpose of our modeling was to understand how compositional, charge, and structural transformations are governed by variations in O/H stoichiometry. The first result we obtained from our computational DFT simulations is that the metastable $P4_{1}$ crystal lattice of the oxygen-poor Y_2_H_4_O composition may present a proper set of active sites for the accommodation of H^+^ cations. In order to identify the possible number of protonic positions, we further investigated how the incorporation of additional amount of oxygen may induce the process of hydrogen exchange in the Y_2_H_4_O system. The model scheme we considered for the simulation of an addition reaction was based on the formal equation Y_2_H_4_O|solid + (1/2)O_2_ -> Y_2_H_3_O(OH)|_solid_. The main feature of the process is that the addition of oxygen (for example, upon treatment with such an oxidizing agent as hydrogen peroxide) results in converting yttrium oxyhydride into the corresponding hydroxyhydride. Evidently, the leading step here is the formation of bridging O^2-^ affecting the interplay of the local lattice geometry and charge states. Under such conversion, when the system combines directly with the additional oxygen atoms, it readily reorganizes the bonding geometry through generation of H^+^ cations in order to keep the overall electro-neutrality for the new resultant composition. In view of chemical energetics, note that the respective enthalpy change of this transformation, $\Delta H_{r}^{0}=-364.7$ kJ/mol, indicates that, unlike the stable $Pmn2_{1}$ and R3m polymorphs of Y_2_H_4_O [28], its $P4_{1}$ chiral modification is highly reactive with respect to oxygen uptake. Moreover, in view of chemical kinetics of crystallization, the choice of such “early” intermediate structure of Y_2_H_4_O as a starting point (i.e., as a precursor) is in full compliance with Ostwald’s scenario of solid-to-solid successive transformations. That is, additional oxidation of the $P4_{1}$ chiral lattice turns out to be favorable because it leads to the formation of a more stable extended composition in which the oxygen atoms are inserted as substitutional ligands of the metal centers within the crystal structure while the $P4_{1}$ crystallographic symmetry remains unchanged.

Figure 1 presents more details on electron transfer and the corresponding change of bonding/geometry configuration. Their interplay gives rise to structural patterns associated with the on-site accommodation of protons. It is also seen that a route to Y_2_H_3_O(OH) proceeds via distribution of the incorporated oxygen over empty interstitial voids of the $P4_{1}$ lattice. The maximum number of possible protonic sites (orbits) is equal to 1 because, in the packing motif of single-crystal architecture of the bulk Y_2_H_4_O, each oxygen is connected to two metal centers. This fact is well illustrated by the elongated bonding distance of 2.454 Å which Y establishes with hydridic hydrogens occupying the H(4) position. Hence, the weakness of the long Y−H(4) connections observed in Y_2_H_4_O positively affects the accessibility of H(4) as a potential protonic site during the oxygen incorporation. In fact, because of a stronger affinity of yttrium for oxygen, the additional oxygen plays a role as the stabilizing agent, thus providing increase in stability. Being combined with the metal center, it behaves as a radical anion that initiates the net hydrogen transfer by breaking the Y−H(4) bond, subsequently repelling a hydride ion from the yttrium site. Since this oxygen (residing in the O(2) position) becomes almost completely negatively charged, it exhibits a higher tendency to combine with the H^+^ cation to form the standard OH^-^ ion. Note further that, as seen in Figure 1, the displacement of the H(4) proton accompanies no noticeable reorganization of internal coordinates of the host lattice. In particular, estimating the hydrogen shift with respect to its initial position H(4) in the precursor Y_2_H_4_O gave a value of 0.947 Å, which is very close to the O−H bonding distance. This implies that, by establishing the O−H ionic connection, the O(2) oxygen tightly holds protonic hydrogen in at H(4), thus protecting the ionized state as the H^+^ cation from reduction and elimination or merging. Moreover, from the symmetry point of view, one can suggest that crystallographic symmetry places some limitations on the possibility of H^-^/H^+^ coexistence in an inorganic crystalline solid. The corresponding rule could be worded as follows: the partial H^-^ → H^+^ on-site conversion may afford subsequent displacement of the generated proton only over lattice positions matching the lowest site-symmetry. Obviously, the crystal systems described by chiral groups are the most relevant for this case, i.e., when H migration proceeds through its spontaneous reconnection from Y to O.

### 2.2. Crystal Structure

The description of the $P4_{1}$ crystal structure of Y_2_H_3_O(OH) is summarized in Table 1 and Table 2, and schematic illustrations are presented in Figure 2. The unit cell contains eight independent atoms which occupy 4a orbits of the lowest point symmetry $C_{1}$. The arrangement of oxygen atoms matches two independent sites: O(1) surrounded tetrahedrally by four yttrium atoms and O(2) which is connected with two yttrium atoms. Y(1) coordinates with O(1) and three hydrogens, H(1)–H(3); its connection with the nearest Y(2) serves as a bridging unit in the [H_3_Y_2_O]^+^ cation. The periodic arrangement of [H_3_Y_2_O]^+^ underlies the 3D cationic framework in which [H_3_Y_2_O]^+^ shares the O(2)−H(4) coupling. The Y−O bond lengths vary from 2.224 to 2.508 Å (Table 2), where the largest values relate to the preferred coordination that O(2) chooses with yttrium. In a set of Y−O connections, O(1) forms a standard bridge between two nearest metal centers. The other oxygen, O(2), puts together the H(4) site and the metal center. Four hydrogen sites, H(1)–H(4), are filled in such a way that the hydrogen anions continue to share the “pristine" proximal ligand positions H(1)–H(3), while the new H(4) position is opened to be occupied by the released hydrogen. The interesting feature is the strong repulsive interaction between the H(1)–H(3) hydrogen anions: the lattice geometry does not allow them to approach one another closer than 2.32 Å. This implies that, according to Switendick’s criterion [31], these negative-charged hydrogens can be considered completely separated from each other. The other interesting feature of the Y_2_H_3_O(OH) structure is that spatial separation of two oppositely charged hydrogen centers is accomplished by displacing the oxygen anions O(2) in order to fix the proton H^+^ in the H(4) position and to form the hydroxide anion OH^-^ (the ion pairing effect). Note that the difference in the behavior of O(1) and O(2) atoms represents a specific example of chemical selectivity which provides the conversion of the oxyhydride host into a hydroxyhydride system.

In the crystallographic sense, the $P4_{1}$ group is minimalistic as compared to the properties of the most other space groups because it presents only a single Wyckoff set of equivalent locations, which comprises of a 4a point orbit. A rapid survey of crystal structures showed that inorganic materials that adopt the tetragonal $P4_{1}$ symmetry are rather scarce. Nevertheless, one can directly refer to the family of compounds A_4_LiH_3_(XO_4_)_4_ (A = K, Rb, NH_4_ and X = S, Se) which crystallize in the $P4_{1}$ structure (e.g., that in Reference [32] and references therein).

### 2.3. Potential of Structural and Thermal Stabilities

We investigated the structural stability of Y_2_H_3_O(OH) to ensure that the crystal lattice is far from dynamical instability. An analysis showed that all the necessary criteria are fulfilled: First, the stability of the tetragonal phase against the relative displacement of sublattices is provided by the positive values of the squares of zone-centered vibrational modes (See Supplementary Materials Table S4. Second, the stiffness matrix (the elasticity tensor) is completely positive definite (Table S8 of SI). This fact confirms the macroscopic stability of the crystalline medium in terms of the elastic energy. The knowledge of elastic constants allowed us to estimate the aggregate characteristics (Table 3).

It is easy to verify that the structural model of Y_2_H_3_O(OH) exhibits macroscopic elastic properties which are typical for ion-covalent crystals except for one peculiarity: the elasticity tensor components, $C_{11}$, $C_{12}$, and $C_{44}$, are close to those values that obey the Cauchy relations for an isotropic cubic medium: $C_{11}=3C_{12}$ and $C_{12}=C_{44}$. Moreover, the indexes $A^{U}$ and $A^{L}$ and the differences $1-C_{33}/C_{11}$ and $1-C_{12}/C_{66}$ indicate a rather low level of the elastic anisotropy. Since in a tetragonal crystal the bulk and shear moduli are directly engaged in the elastic behavior, such weak anisotropy suggests that a purely pairwise and directional character of the interatomic Coulomb forces is the distinguishing feature of the bonding situation in Y_2_H_3_O(OH). Note that a number of cubic binary and even ternary ionic compounds demonstrate a similar bonding picture. However, when the tetragonal distortion lowers the initial cubic symmetry, in the low-symmetry phase, the bonding forces acquire an angular character via indirect many-particle contributions caused by electron–ion (or electron–electron) interactions. This leads to competition between central and noncentral forces which may violate the Cauchy relations. If the effect of symmetry reduction is marginal, i.e., the lattice distortion does not produce or increase an overlap of the ionic cores, the renormalized charge distributions around the ion centers should remain localized to hold the direct/central character of the interaction between ions. To demonstrate that this particular factor underlies the weak anisotropy in Y_2_H_3_O(OH), we analyzed a topology of valence charge partitioning in terms of the electron localization function (ELF).

The comparison of the different ELF visualizations is presented in Figure 3. The characteristic feature clearly seen in the limit ELF→1 is the global separation into electron-rich and electron-deficient spatial regions. In particular, this configuration reflects the exceptionally high level of localization of valence electron density at the H^-^ centers.

Utilization of the AIM protocol for the theoretical charge densities provided us with the Bader charges summarized in Table 4. These values can be compared with the charge configuration of the nominal ionic model with a mixed-anion order: [Y_2_^3+^H_3_^-1^O^2-^(OH)^-1^]. It is seen that the bonding situation is to a certain extent not complicated because the overall bonding character is determined by the interplay of ionic and covalent connectivities. Since the chiral lattice of Y_2_H_3_O(OH) belongs to the polar crystal class 4, we also examined how strongly the outer electron shells are distorted due to the coupling of valence electrons with dipole-active optical vibrational modes. The vibration-assisted enhancement of charge states was characterized in terms of principal values of the Born dynamical charge (Table 4). Following the model of formal charge partitioning, one can see that the dynamical charge redistribution strengthens the yttrium donating ability, i.e., the most positive charges tend to be concentrated on the pair of yttriums while the most negative local charges are shared by the oxygen, O(1), and the three hydrogens, H(1)–H(3). In fact, we are dealing here with the cooperative effect of electron polarization which gives rise to dynamical enhancement of the valence charge localization.

The evaluation of the ground-state formation energy (shown in the last raw of Table 1a) indicates that the bulk structure of Y_2_H_3_O(OH) represents an enthalpically stable condensed phase. Based on the standard dehydration scheme for a hydroxide solid, we have verified the stability of Y_2_H_3_O(OH) with respect to the decomposition into the mixture of oxide, hydride, and water. Since the estimates have shown that the negative enthalpy difference may be small, −0.1÷−0.3 eV/(f.u.), (i.e., the structure is still more stable at the zero temperature than the mixture of yttria, hydride, and water), we have further examined what may happen when the system is subjected to external heating. Accordingly, the principally important question we considered is how stable is the predicted crystal structure against the action of heat. In order to probe a destabilization role of thermal treatment and, correspondingly, to evaluate the thermal stability of the $P4_{1}$ chiral phase, we performed a series of ab initio molecular dynamics (MD) simulations.

Shown in Figure 4 are thermal motions of ions which are presented in terms of temperature evolution of the pair distribution function (PDF). It is seen that the first, second, and third peaks are intense and narrow; their high values reflect strong cation–anion interactions. The dynamical evolution of the peaks with respect to their broadening, displacements, and fluctuations shows insignificant temperature-dependent changes. Thus, these simulations indicate the smallness of the thermodynamic driving force to initiate the thermal dehydration and other thermal effects associated with the atomic disorder. Hence, the crystal lattice of Y_2_H_3_O(OH) remains robust against heating up to 400 K. Moreover, the theoretical estimate of the Debye temperature (a marker of the thermal behavior), $\Theta_{D}=495$ K, and the Grüneisen parameter (a marker of the thermal expansion), γ=1.87 made by using the elastic parameters of Table 3 demonstrate values typical for solid hydroxides.

### 2.4. Dihydrogen Bonding

As it follows from the protonation chemistry of transition metal hydride complexes [33], a dissociative pathway of metal–hydrogen bond may lead to the formation of dihydrogen bonding patterns. We have tested whether tentative signs of hydrogen bonding can be seen from vibrational analysis of Y_2_H_3_O(OH). In particular, we have found that the decomposition of a set of relatively low-lying frequencies (Table S4 of SI), 1008–1393 cm${}^{-1}$, which are associated with the stretching movements among hydrogen pairs, points to hydrogen bonding in the H(1)–H(4) and H(2)–H(4) contacts of hydridic and protonic hydrogens. On the other hand, the most high-frequency modes, 3336 and 3340 cm${}^{-1}$, can be assigned to the decoupled stretching distortions of the OH^-^ anion (similarly to solid hydroxides). We have also assumed that the large value of the low-frequency shift, *ca* 350 cm${}^{-1}$, in the spectral behavior of proton donors in Y_2_H_3_O(OH) (calculated as the difference between high-frequency stretching vibrations in Y_2_H_3_O(OH) and Y(OH)_3_) may serve as the other good signature of hydrogen bonding.

The structural grouping and geometry of the system of hydrogen bonds are described in Table 5 and depicted in Figure 5 in terms of H···H characteristic contacts. The results demonstrate that, by joining yttrium polyhedral units in the crystal lattice, the non-covalent coupling between the OH protons and hydride anions establishes a robust network of dihydrogen bonds [33,34,35,36]. On the base of hydrogen equilibrium positions (Table 1b), the strength and directionality of the dihydrogen bonds in Y_2_H_3_O(OH) can be characterized as follows. Of two H···H short contacts, the smallest, 1.753 Å for the H(1)···H(4) distance, is the most representative. The other H(2)···H(4) contact elongated to 2.050 Å also belongs to a typical dihydrogen bond [37]. Both contacts may be characterized as strong and almost strong because the distances observed are significantly smaller than the sum of van der Waals radii for hydrogen. Accordingly, the third H(4)···H(3) contact longer than 2.4 Å cannot be classified as a dihydrogen bond. We note further that strong bending of the O−H···H angle, which varies between $114{\;}^{\circ }$ and $168{\;}^{\circ }$ with $141{\;}^{\circ }$ as an average, is a direct indication that the dihydrogen bond is realized as the O−H···σ interaction in full accord with the theoretical picture presented in Reference [38]. We believe that the equilibrium distribution of dihydrogen bonds in Y_2_H_3_O(OH) cannot be easily destroyed because our MD simulations have clearly shown that the dihydrogen bonds are able to withstand the impact of thermal heating. The role of dihydrogen bonds is that, by linking together the [H_3_Y_2_O] chains via OH groups, they provide the reinforcement of the lattice structure of Y_2_H_3_O(OH). In other words, the dihydrogen bond represents an important structural element [37] contributing to the stability of the chiral geometry of Y_2_H_3_O(OH).

One geometric characteristic of the H(1)H(4)H(2) symbolic triangle is the proximity of the H(1) and H(2) sites, which, in terms of the H(1)···H(2) contact length (2.41 Å), is very close to the van der Waals diameter (e.g., Figure 5). The rationalization of this fact is interesting in the sense that the strong dihydrogen bonding tends to limit the electrostatic repulsion between the nearest, similarly charged hydridic hydrogens by placing them on the distance of closest approach. In the context of the bonding geometry, such an accommodation becomes most favorable because the proton at the H(4) position gains a possibility to simultaneously attacks the two closely located hydride centers H(1) and H(2).

### 2.5. Y_2_H_3_O(OH) as a Chiral Crystal

Y_2_H_3_O(OH) crystallizes entirely in a chiral structure in which all the atoms of the unit cell occupy the 4a crystallographic sites (Table 1b). That is, the structural organization is provided by the four-atom patterns belonging to one orbit. Since the site symmetry is described by a chiral (pure rotational) $C_{1}$ point group, a rotational atom stacking in the pattern leads to the formation of α-helices corresponding to 1d-helical chains decorated by Y(1), Y(2), O(1), O(2), H(1), H(2), H(3), and H(4) atoms. Accordingly, side-by-side spatial arrangements of all of them form a chiral framework in Y_2_H_3_O(OH) which consists of 8 single helices.

As shown in Figure 6, the helical assembly comprises the right-handed helical chains around the crystallographic screw axis $4_{1}$, which spreads along the *z*-direction of the entire structure. Interestingly, the helical organization affords a tolerable amount of freedom because some of the different 4a atomic positions can be grouped to compose joined helical chains (Figure 6e). In this context, one could associate the high degree of structural orderings in the tetragonal phase with the formation of rotational stacking arrangements of the spatially separated OH^-^ anions. That is, in order to enforce itself via dihydrogen bonding, the lattice has to rotate relative to the oxygen fixed positions within the only possible tubular orientation around the *z* axis.

### 2.6. Electron Band Structure

The electron structure calculations showed that the bulk Y_2_H_3_O(OH) is a direct-gap semiconductor with the fundamental band-gap width of 3.4 eV in the Γ point. This value is confirmed by the estimate, 3.6 eV, which was obtained within the $G_{0}W_{0}$ approximation (Figure S9 of SI). The density of states plots are shown in Figure 7a,b. A comparison indicates the prevailing contribution of the hydride anion *s* electron states in the valence band region near the Fermi energy. This is due to the strong localization of electrons at the hydride anion. One sees large peaks of DOS in the wide interval of the valence band, in which the behavior of *p* oxygen states mimics the hydrogen *s* states. However, the contribution of O(2) oxygen is narrowed and shifted by *ca* 2 eV, as compared with the corresponding contribution of O(1). The other feature is that the conduction band, which is formed by contributions of the *s* empty states of all the elements, is significantly broadened along the energy axis. Figure S5 of SI indicates that the bands with s-electron character are relatively well dispersed. The calculated effective masses shown here characterize anisotropy and dispersion of the electron bonds near the Γ point along the Γ→Z and Γ→X high-symmetry directions.

### 2.7. Optical Responses

Inorganic materials with the spatial chirality are of great interest for various optical applications. The structural model of Y_2_H_3_O(OH) corresponds to a uniaxial chiral (right-handed) material which is optically active and exhibits optical anisotropy governed by the tetragonal axis. As shown in Figure 8a and in Figures S6–S8 of SI, our calculations revealed anomalous dispersion visible at short wavelengths up to *ca* 370 and 420 nm for ordinary and extraordinary rays, respectively.

The large difference in behavior of these light rays along the whole spectrum has a pronounced impact on the magnitude of $n_{e}-n_{o}$. This may lead to a scenario in which the chiral material exhibits new experimentally undiscovered effect of anomalous birefringence.

Nonlinear optical (NLO) properties are more sensitive to the details of the electron band structure and to selection rules. In the crystalline medium without inversion center, the asymmetry of the electron transitions gives rise to such NLO phenomenon of the coherent nature as the second-harmonic generation (SHG) [39,40]. We considered a possibility of frequency doubling in terms of the second-order response of Y_2_H_3_O(OH) on the electric field of the incident light wave. Shown in Figure 8b and in Figures S10 and S11 of SI is the theoretical prediction for the $\chi_{zzz}\left(2\omega ,\omega ,\omega \right)$ component of the SHG susceptibility tensor. According to our calculations, this component determines a dominant fraction of the bulk SHG in Y_2_H_3_O(OH); tensor components have been estimated in the ratio as $\chi_{333}^{\left(2\omega \right)}$ : $\chi_{113}^{\left(2\omega \right)}$ ∼5.8 : 1. The theoretical assessment for photon energies corresponding to λ=400 and 422 nm provided the evaluation of $\chi_{333}^{\left(2\omega \right)}$ as 26.4 and 23.6 pm/V, respectively. These values of nonlinear coefficients are close to the NLO susceptibilities typical for perovskite ferroelectrics with strong optical nonlinearities [41].

### 2.8. The Role of Substitutions

Given that the chiral structure of Y_2_H_3_O(OH) is stable, one can ask whether the change of yttrium may keep crystallization in the $P4_{1}$ lattice. In other words, whether the full cation exchange can afford a whole family of stoichiometric hydroxyhydrides M_2_H_3_O(OH) (where M standing on the yttrium cation site relates to a three-valence metal element) with the multianion ratio 3:1:1.

To resolve this issue, we have evaluated the structural stability of three systems differing in yttrium substitution by M = Sc, La, and Gd (Tables S5–S7 of SI). The results shown in Table 6 and in Tables S1–S3 of SI demonstrate that the entirely substituted hydroxyhydrides crystallize in the tetragonal phase with the $P4_{1}$ chiral crystal structure. All the materials exhibit relatively similar crystallographic c/a ratios close to 1.5. Calculated X-Ray diffraction patterns for predicted M_2_H_3_O(OH) are shown in Figures S1–S4 of SI. The valence count shows that the compounds will behave as band insulators. Based on the smooth performance of the hydrogens which contribute to assembly of the helical structures in Y_2_H_3_O(OH), one can further suggest that the similar scheme of hydrogen bonding exists in M_2_H_3_O(OH), thus making the strong dihydrogen bonding a general feature of these chiral systems (Table 6b). The comparison of Table 5 and Table 6b indicates that both the interaction strength and the proton accepting/donating abilities are roughly the same for the whole range of hydroxyhydride systems predicted in the present work.

### 2.9. Discussion

In summary, by modeling the oxidation process of the hydride host in terms of different structural combinations of metal–oxygen and oxygen–hydrogen interactions, we have predicted a novel class of inorganic crystalline materials—hydroxyhydrides which can be described by the chemical formula M_2_H_3_O(OH) with the cation site M occupied by the trivalent metal element. Based on the results of structure-modeling simulations, we believe that M_2_H_3_O(OH) may represent a wider range of stoichiometric compounds than those four (M = Y, Sc, La, and Gd) that have been reported in the present work. It is important to emphasize that a major common trait of these mixed-anionic systems is the absolute chirality determined by the $P4_{1}$ crystal structure. In the Y_2_H_3_O(OH) case study, we have investigated a number of structural and bonding features such as the extra-high localization of valence charge densities, the strong dihydrogen bonding, and the stability of the spatial arrangement of hydridic and protonic hydrogens in the lattice. The effect of strong localization corresponds to a specific charge ordering connected with the chiral organization of the metal cations and the anions which are standing in positions that form helical curves spreading along the tetragonal axis. The effect of twisting of the H^-^ and H^+^ hydrogens related to different chains causes their linking by dihydrogen bonds. In the context of structure–property relationships, valuable insights into elastic, electronic, and optical properties of the bulk Y_2_H_3_O(OH) have been gained.

In the context of application-oriented research, one can emphasize that our findings open a promising route to the development of novel relatively simple chiral materials for possible applications in optoelectronics, laser, and nonlinear optics. The development of inorganic materials with NLO properties is one of the important challenges. However, the problem is that some materials with desirable NLO properties can be formed under extreme synthesis conditions that are not so easy to reach. The organic compounds that may exhibit NLO properties under normal conditions cannot withstand external effects such as high electromagnetic fields, etc. In light of this, Y_2_H_3_O(OH) has great potential to overcome a number of current challenges and limitations.

## 3. Methods

### 3.1. Computational Details

We performed a series of electron structure calculations within the framework of density functional theory (DFT) and Hedin’s approximation in the GW method [42] by using Vienna ab initio simulation package [43,44] (VASP) with the potential projector augmented-wave method [45,46] (PAW).The PAW-PBE pseudo-potentials involved the plane-wave basis sets of $4s^{2}4p^{6}5s^{2}4d^{1}$, $2s^{2}2p^{4}$, and $1s^{1}$ valence electron configurations for the Y, O, and H elements, respectively. The plane-wave energy cutoffs of 700 eV have been used to provide well-converged free-energy results in the periodic calculations with the degree of accuracy below 1 meV/(unit cell). The Kohn–Sham one-electron eigenstates and energies were deduced on the base of Perdew–Burke–Ernzerhof (PBE) GGA exchange-correlation functional [47]. The equilibrium geometries were fully optimized in PBE-GGA with respect to cell parameters and internal atomic positions. To take into account the effect of nonlocality contributions, we calculated the electron structure within the range-separated Heyd–Scuseria–Ernzerhof (HSE06) hybrid functional formalism [48,49,50,51,52].According to References [53,54], the amount of Fock exchange in the hybrid functional has been modeled via a material-dependent parameter—the inverse macroscopic electronic constant, $\epsilon_{\infty }^{-1}$. Using the average value $\epsilon_{\infty }=4.8$, estimated from the numerical procedure of density functional perturbation theory [55],the relevant fraction of the Fock exchange in the HSE06 functional was adjusted to 0.21. A 2×2×2 supercell of 256 atoms was employed to verify the thermal stability of the bulk Y_2_H_3_O(OH) by using a NVE molecular dynamics (MD) simulations, as implemented in VASP.

The optical response of the bulk Y_2_H_3_O(OH) in terms of quasiparticle energies were evaluated within the computational scheme for the GW routines as implemented in VASP. The energies of the single quasi-particle states have been calculated via multi-shot series of the zeroth order in the self-energy G${}_{0}$W${}_{0}$. The optical properties were deduced from the G${}_{0}$W${}_{0}$ results in terms of absorption, refractivity, and reflectivity coefficients. The nonzero independent elements, $\chi_{113}^{\left(2\omega \right)}$ and $\chi_{333}^{\left(2\omega \right)}$, of the second-order nonlinear susceptibility tensor (second-harmonic generation) were estimated by using the procedure of Reference [56] implemented in an all-electron full-potential linearized augmented-plane wave code Elk [57]. Since we were interested in the overall spectral dependence of the nonzero elements of the second-harmonic susceptibility tensor, the many-particle effects were introduced in the “scissors” approximation.

### 3.2. Post-Processing Analysis

Three descriptors have been employed for the detail processing of theoretical charge densities. First, the charge-density distributions were treated in terms of numerical procedures [58,59], implementing the real-space technique (QTAIM) of a grid-based Bader analysis [60]. Second, a topology of the valence charge densities was classified in terms of the electron localization function [61] (ELF). Third, the contribution of many-body polarization effects (i.e., the dynamics of the polar character of chemical bonds) was characterized in terms of the Born dynamical charges [62]. The relevant patterns of group–subgroup relations were constructed by means of the program tools [63,64] hosted by Bilbao Crystallographic Server [65,66,67,68].The ISOTROPY software suite [69,70] and the VESTA program [71] were used in the course of evaluation of the crystal structures and electron topologies. Also, the structural and ELF visualizations were made by means of the VESTA program. The formation energy (the heat of formation at T=0 K) was evaluated as the difference between the total energy per formula unit and the sum of the energies of constituent elements.

### 3.3. Crystal Assembly in Terms of Structural Transformations

In the oxidation of yttrium hydrides, because of a stronger affinity of elemental yttrium for oxygen and its preference to exhibit 3+ oxidation state, there appears the concurrency between the incorporated oxygen and host hydrogen for connectivity with the metal center. Since the metal prefers to bind the oxygen atom, the oxidization process of the hydride system is accompanied by two factors: (i) the change of initial valence electron configurations proceeding in terms of delocalization-localization conversion and (ii) a chain of structural transformations leading to a stable condensed phase [72]. One can, therefore, suggest that, when H^-^ is topochemically replaced by oxygen, the interplay of processes, such as the Y−H covalent bond cleavage and the Y−O ionic bond formation, may keep a certain part of the interstitial hydrogen species in the lattice.

The conceptual methodology with applications to various compounds was described in previous works [28,73,74,75]. Based on this methodology, we modeled an assembly of the crystalline Y_2_H_3_O(OH) by considering the formal oxygenation/dehydrogenation of the yttrium–hydrogen system in terms of the interplay between chemical composition and lattice architecture. A set of structural modifications was presented through possible (intermediate) lattice geometries, which were generated by a stoichiometric distribution of inserted oxygen atoms over the different crystallographic voids. The chemical reactivity was deemed through a partial cleavage of metal hydride bonds along with subsequent formation of metal–oxygen bonds. The $Fm\bar{3}m$ crystalline superstructure was chosen as a trial template because its high (archetypal) cubic symmetry affords a maximal set of concurring geometries and packings that may span various oxygen distributions over different composition-lattice-packing configurations. Final screening of candidate structures was performed on the base of the Bärnighausen tree [76,77] for two sequences of lattice transformations—$Pn\bar{3}m⟹P4_{1}$ and $P4_{2}/mcm⟹P4_{1}$—reflecting the ways along which a crystal symmetry is lowering.

### 3.4. Elastic Analysis

The Laue class 4 of the tetragonal phase retains seven independent elastic constants [78], which in Voigt notation are $C_{11}$, $C_{12}$, $C_{13}$, $C_{33}$, $C_{16}$, $C_{44}$, and $C_{66}$. To analyze the elastic properties of the material, the ELATE online tool [79,80] was used. The elastic moduli were evaluated within the Voigt=-Reuss–Hill averaging approach [81].To quantify the anisotropy of the elastic behavior of Y_2_H_3_O(OH), the relative (the universal anisotropy index $A^{U}$) and absolute ($A^{L}$) measures of anisotropy were estimated according to relations given in References [82] and [83], respectively, since for the completely isotropic body $A{}^{U}=A{}^{L}\;\equiv \;0$, nonzero values of the indexes $A^{U}$ and $A^{L}$ determine the magnitude of the elastic anisotropy. The longitudinal elastic anisotropy for the tetragonal body was estimated via the relation $C_{33}/C_{11}$. The interplay of elastic and plastic properties underlies the hardness of a system. This characteristic was evaluated in terms of the Vickers hardness, $H_{V}$, by using two semi-empirical model relations proposed in References [84,85].

## Figures and Tables

**Table 1:** Equilibrium crystal data predicted for Y_2_H_3_O(OH).

Parameter	Value
Composition	Y_2_H_3_O(OH)
Structure type	chiral
Crystal system	tetragonal
Space group	$P4_{1}$ (76)
*a*, Å	6.049
*c*, Å	9.264
c/a	1.531
*V*, Å${}^{3}$	338.9
Number of f.u. per cell, *Z*	4
Theoretical density, g/cm${}^{3}$	4.19
Formation energy, kJ/mol	−1172.8

**a** Main crystallographic data.

**Table:** 

Atom	Wyckoff	Point	x	y	z
	**position**	**symm.**			
Y(1)	4a	1	0.31442	0.20398	0.20737
Y(2)	4a	1	0.78673	0.30972	0.04096
O(1)	4a	1	0.42739	0.35814	0.00094
O(2)	4a	1	0.18891	0.31293	0.45537
H(1)	4a	1	0.62524	0.01309	0.11845
H(2)	4a	1	0.07735	0.46495	0.13807
H(3)	4a	1	0.10548	0.06222	0.00000
H(4)	4a	1	0.26778	0.18964	0.50260

**b** Atomic positions.

**Table 2:** Equilibrium interatomic distances (Å) in Y_2_H_3_O(OH).

H(1)−Y(1)	H(1)−Y(2)	H(2)−Y(1)	H(2)−Y(2)	H(3)−Y(1)	H(3)−Y(2)	H(4)−O(2)	O(1)−Y(1)	O(1)−Y(2)	O(2)−Y(1)	O(2)−Y(2)
2.355	2.165	2.227	2.187	2.388	2.470	0.987	2.235	2.224	2.508	2.421
	2.141	2.274			2.472		2.431	2.249		

**Table 3:** Summary of the aggregate characteristics calculated for Y_2_H_3_O(OH) in terms of the elasticity tensor ($C_{ij}$), the bulk (*B*), shear (*G*), and Young’s (*E*) moduli (in GPa): The relation G/B is Pugh’s ratio, ν denotes Poisson’s ratio, and the indexes $A^{U}$ and $A^{L}$ describe the elastic anisotropy. Estimates of the Vickers hardness are given via $H_{V}$ (in GPa). The longitudinal elastic anisotropy ratio $C_{33}/C_{11}\;≃\;0.97\;$.

$C_{11}$	$C_{12}$	$C_{13}$	$C_{33}$	$C_{16}$	$C_{44}$	$C_{66}$	*B*	*E*	*G*	G/B	ν	$A^{U}$	$A^{L}$	$H_{V}$
123.3	45.2	31.5	120.0	−7.3	50.8	43.9	64.6	111.1	45.8	0.71	0.21	0.13	0.056	9.5/9.3

**Table 4:** Characteristics of ions in Y_2_H_3_O(OH) in terms of effective charges (in units of $\left|e\right|$): The quantities $Q_{B}\left(M\right)$ describe the Bader effective charges. $Z_{\mathrm{ij}}^{*}$ is the matrix of the Born dynamical charges. The quantity ${\bar{Z}}^{*}\left(M\right)$ corresponds to the average of the principal values over crystal axes. The proton occupying the H(4) position exhibits a positive effective charge of +0.53|e|, typical for charge distribution in OH^-^.

M	Y(1)	Y(2)	O(1)
$Q_{B}\left(M\right)$	+2.06	+2.01	−1.35
$Z_{\mathrm{ij}}^{*}\left(M\right)$	$\left(\begin{array}{ccc} +3.05 & 0 & 0\\ 0 & +3.75 & 0\\ 0 & 0 & +3.54 \end{array}\right)$	$\left(\begin{array}{ccc} +3.92 & 0 & 0\\ 0 & +2.74 & 0\\ 0 & 0 & +3.41 \end{array}\right)$	$\left(\begin{array}{ccc} -2.07 & 0 & 0\\ 0 & -2.82 & 0\\ 0 & 0 & -2.82 \end{array}\right)$
${\bar{Z}}^{*}\left(M\right)$	+3.45	+3.36	−2.57
M	O(2)	H(1)/H(2)/H(3)	H(4)
$Q_{B}\left(M\right)$	−1.33	−0.627/−0.636/−0.648	+0.533
$Z_{\mathrm{ij}}^{*}\left(M\right)$	$\left(\begin{array}{ccc} -1.87 & 0 & 0\\ 0 & -1.96 & 0\\ 0 & 0 & -1.88 \end{array}\right)$	$\left(\begin{array}{ccc} -0.81 & 0 & 0\\ 0 & -1.07 & 0\\ 0 & 0 & -0.91 \end{array}\right)$	$\left(\begin{array}{ccc} +0.48 & 0 & 0\\ 0 & +0.55 & 0\\ 0 & 0 & +0.55 \end{array}\right)$
${\bar{Z}}^{*}\left(M\right)$	−1.90	−0.93	+0.53

**Table 5:** Canonical proof of dihydrogen bonding in Y_2_H_3_O(OH) in terms of hydroxid–hydride bond connectivities. The last column shows the bond–charge correlation: the difference $\delta Q_{B}\left(H\right)$ means the characteristic loss of charge of the hydride anion (in units of $\left|e\right|$) participating in the dihydrogen bonding with respect to the effective charge on noninteracting H(3).

O-H···H bond	O-H···H, Å	O···H, Å	∠O-H···H	$\delta Q_{B}\left(H\right)$
O(2)−H(4)···H(1) bond	1.753	2.727	$168{\;}^{\circ }$	0.021
O(2)−H(4)···H(2) bond	2.050	2.608	$114{\;}^{\circ }$	0.012
O(2)−H(4)···H(3) bond	2.724	2.914	$124{\;}^{\circ }$	-

**Table 6:** Crystal chemistry of substituted hydroxyhydrides M_2_H_3_O(OH).

Parameter/Value			
Composition	Sc_2_H_3_O(OH)	La_2_H_3_O(OH)	Gd_2_H_3_O(OH)
Structure type	chiral	chiral	chiral
Crystal system	tetragonal	tetragonal	tetragonal
Space group	$P4_{1}$ (76)	$P4_{1}$ (76)	$P4_{1}$ (76)
*a*, Å	5.656	6.460	6.121
*c*, Å	8.717	9.645	9.330
c/a	1.541	1.493	1.524
*V*, Å${}^{3}$	278.9	402.5	349.5
Number of f.u.	4	4	4
per cell, *Z*			
Theoretical	3.00	5.18	6.66
density (g/cm${}^{3}$)			
Formation	−1091.2	−1160.0	−1160.8
energy (kJ/mol)			

**a** Main crystallographic data.

**Table:** 

O−H···H	O−H···H	O···H	∠O−H···H
bond	(Å)	(Å)	
Sc_2_H_3_O(OH)			
O(2)−H(4)···H(1)	1.610	2.595	$172{\;}^{\circ }$
O(2)−H(4)···H(2)	1.998	2.477	$107{\;}^{\circ }$
La_2_H_3_O(OH)			
O(2)−H(4)···H(1)	1.849	2.791	$158{\;}^{\circ }$
O(2)−H(4)···H(2)	2.074	2.751	$124{\;}^{\circ }$
Gd_2_H_3_O(OH)			
O(2)−H(4)···H(1)	1.786	2.754	$166{\;}^{\circ }$
O(2)−H(4)···H(2)	2.036	2.628	$116{\;}^{\circ }$

**b** Structural description of dihydrogen bonding in terms of hydroxid–hydride bond connectivities.

## Supplementary Materials

The supplementary materials are available online at https://www.mdpi.com/1996-1944/13/4/994/s1, Table S1: Sc_2_H_3_O(OH); Table S2: La_2_H_3_O(OH); Table S3: Gd_2_H_3_O(OH); Table S4: Y_2_H_3_O(OH); Table S5: Sc_2_H_3_O(OH); Table S6: La_2_H_3_O(OH); Table S7: Gd_2_H_3_O(OH); Table S8: M_2_H_3_O(OH) (M = Y, Sc, La, Gd); Table S9: Nonzero components of the elasticity for M_2_H_3_O(OH) (M = Sc, La, Gd) in GPa; Table S10: Summary of aggregate parameters calculated for M_2_H_3_O(OH) (M = Sc, La, Gd); Table S11: Fulfilment of the Born stability conditions1 for the tetragonal phase of M_2_H_3_O(OH) (M = Y, Sc, La, Gd); Table S12: Components of the second-order susceptibility tensor of Y_2_H_3_O(OH) evaluated for different photon wavelengths; Table S13: Y(OH)_3_; Figure S1: X-ray diffraction pattern for the Y_2_H_3_O(OH) structure; Figure S2: X-ray diffraction pattern for the Sc_2_H_3_O(OH) structure; Figure S3: X-ray diffraction pattern for the La_2_H_3_O(OH) structure; Figure S4: X-ray diffraction pattern for the Gd_2_H_3_O(OH) structure; Figure S5: The total density of states, band structure and effective masses of Y_2_H_3_O(OH); Figure S6: Real and imaginary part of dielectric function of the Y_2_H_3_O(OH); Figure S7: Spectral behavior of extinction index in Y_2_H_3_O(OH); Figure S8: Spectral behavior of reflection (R) and transmission (T) in Y_2_H_3_O(OH); Figure S9: The Tauc plot for Y_2_H_3_O(OH); Figure S10: Second-harmonic generation (SHG) spectrum of Y_2_H_3_O(OH) represented in terms of the spectral behavior of tensor component χxxz(2ω, ω, ω); Figure S11: Second-harmonic generation (SHG) spectrum of Y_2_H_3_O(OH) represented in terms of the spectral behavior of tensor component χzzz(2ω, ω, ω).

## References

[1] Wu C., Lin L., Jiang X., Lin Z., Huang Z., Humphrey M.G., Halasyamani P.S., Zhang C. (2019). K5(W3O9F4)(IO3): An Efficient Mid-Infrared Nonlinear Optical Compound with High Laser Damage Threshold. Chem. Mater..

[2] Tang J., Liang F., Meng X., Kang K., Zeng T., Yin W., Xia M., Lin Z., Kang B. (2019). Two in one: An unprecedented mixed anion, Ba_2_(C_3_N_3_O_3_)(CNO), with the coexistence of isolated sp and sp2 *π*-conjugated groups. Dalton Trans..

[3] Oreshonkov A.S., Roginskii E.M., Atuchin V.V. (2020). New candidate to reach Shockley–Queisser limit: The DFT study of orthorhombic silicon allotrope Si(oP32). J. Phys. Chem. Solids.

[4] Juhwan N., Jaehoon K., Helge S.S., Lengeling B.S., Gregoire J.M., Aspuru-Guzik A., Yousung J. (2019). Inverse Design of Solid-State Materials via a Continuous Representation. Materials.

[5] Agar J.C., Pandya S., Xu R., Yadav A.K., Liu Z., Angsten T., Saremi S., Asta M., Ramesh R., Martin L.W. (2016). Frontiers in strain-engineered multifunctional ferroic materials. MRS Commun..

[6] Hayward M.A., Cussen E.J., Claridge J.B., Bieringer M., Rosseinsky M.J., Kiely C.J., Blundell S.J., Marshall I.M., Pratt F.L. (2002). The Hydride Anion in an Extended Transition Metal Oxide Array: LaSrCoO_3_H_0.7_. Science.

[7] Hayward M.A. (2014). Topochemical reactions of layered transition-metal oxides. Semicond. Sci. Technol..

[8] Helps R.M., Rees N.H., Hayward M.A. (2010). Sr_3_Co_2_O_4.33_H_0.84_: An Extended Transition Metal Oxide-Hydride. Inorg. Chem..

[9] Yamamoto T., Kageyama H. (2013). Hydride Reductions of Transition Metal Oxides. Chem. Lett..

[10] Kobayashi Y., Hernandez O., Tassel C., Kageyama H. (2017). New chemistry of transition metal oxyhydrides. Sci. Technol. Adv. Mater..

[11] Uppuluri R., Sen Gupta A., Rosas A.S., Mallouk T.E. (2018). Soft chemistry of ion-exchangeable layered metal oxides. Chem. Soc. Rev..

[12] Fokin V., Malov Y., Fokina E., Troitskaya S., Shilkin S. (1995). Investigation of interactions in the TiH_2_-O_2_ system. Int. J. Hydrog. Energy.

[13] Fokin V., Malov Y., Fokina E., Shilkin S. (1996). Study of the phase-forming features in the ZrH_2_-O_2_ system. Int. J. Hydrog. Energy.

[14] Fokin V.N., Fokina E.E., Shilkin S.P. (2004). Oxidation of Metal Hydrides with Molecular Oxygen. Russ. J. Gen. Chem..

[15] Sakaguchi T., Kobayashi Y., Yajima T., Ohkura M., Tassel C., Takeiri F., Mitsuoka S., Ohkubo H., Yamamoto T., Kim J.E. (2012). Oxyhydrides of (Ca,Sr,Ba)TiO_3_ Perovskite Solid Solutions. Inorg. Chem..

[16] Bang J., Matsuishi S., Hiraka H., Fujisaki F., Otomo T., Maki S., Yamaura J.i., Kumai R., Murakami Y., Hosono H. (2014). Hydrogen Ordering and New Polymorph of Layered Perovskite Oxyhydrides: Sr_2_VO_4–x_H_x_. J. Am. Chem. Soc..

[17] Mizoguchi H., Park S., Hiraka H., Ikeda K., Otomo T., Hosono H. (2014). An Anti CuO_2_-type Metal Hydride Square Net Structure in Ln_2_M_2_As_2_H_x_ (Ln = La or Sm, M = Ti, V, Cr, or Mn). Angew. Chem. Int. Ed..

[18] Park S.W., Mizoguchi H., Hiraka H., Ikeda K., Otomo T., Hosono H. (2017). Transformation of the Chromium Coordination Environment in LaCrAsO Induced by Hydride Doping: Formation of La_2_Cr_2_As_2_O_y_H_x_. Inorg. Chem..

[19] Tassel C., Goto Y., Watabe D., Tang Y., Lu H., Kuno Y., Takeiri F., Yamamoto T., Brown C.M., Hester J. (2016). High-Pressure Synthesis of Manganese Oxyhydride with Partial Anion Order. Angew. Chem. Int. Ed..

[20] Jin L., Lane M., Zeng D., Kirschner F.K.K., Lang F., Manuel P., Blundell S.J., McGrady J.E., Hayward M.A. (2018). LaSr_3_NiRuO_4_H_4_: A 4d Transition-Metal Oxide–Hydride Containing Metal Hydride Sheets. Angew. Chem. Int. Ed..

[21] Katayama T., Chikamatsu A., Kamisaka H., Yokoyama Y., Hirata Y., Wadati H., Fukumura T., Hasegawa T. (2015). Topotactic synthesis of strontium cobalt oxyhydride thin film with perovskite structure. AIP Adv..

[22] Masuda N., Kobayashi Y., Hernandez O., Bataille T., Paofai S., Suzuki H., Ritter C., Ichijo N., Noda Y., Takegoshi K. (2015). Hydride in BaTiO_2.5_H_0.5_: A Labile Ligand in Solid State Chemistry. J. Am. Chem. Soc..

[23] Mongstad T., Platzer-Björkman C., Maehlen J.P., Mooij L.P., Pivak Y., Dam B., Marstein E.S., Hauback B.C., Karazhanov S.Z. (2011). A new thin film photochromic material: Oxygen-containing yttrium hydride. Sol. Energy Mater. Sol. Cells.

[24] Montero J., Martinsen F.A., García-Tecedor M., Karazhanov S.Z., Maestre D., Hauback B., Marstein S. (2017). Photochromic mechanism in oxygen-containing yttrium hydride thin films: An optical perspective. Phys. Rev. B.

[25] You C.C., Moldarev D., Mongstad T., Primetzhofer D., Wolff M., Marstein E.S., Karazhanov S.Z. (2017). Enhanced photochromic response in oxygen-containing yttrium hydride thin films transformed by an oxidation process. Sol. Energy Mater. Sol. Cells.

[26] Nafezarefi F., Schreuders H., Dam B., Cornelius S. (2017). Photochromism of rare-earth metal-oxy-hydrides. Appl. Phys. Lett..

[27] Zapp N., Auer H., Kohlmann H. (2019). YHO, an Air-Stable Ionic Hydride. Inorg. Chem..

[28] Pishtshev A., Strugovshchikov E., Karazhanov S. (2019). Conceptual Design of Yttrium Oxyhydrides: Phase Diagram, Structure, and Properties. Cryst. Growth Des..

[29] DuBois M.R., DuBois D.L. (2009). The roles of the first and second coordination spheres in the design of molecular catalysts for H2production and oxidation. Chem. Soc. Rev..

[30] Hayashi K., Sushko P.V., Hashimoto Y., Shluger A.L., Hosono H. (2014). Hydride ions in oxide hosts hidden by hydroxide ions. Nat. Commun..

[31] Switendick A.C. (1979). Bandstructure calculations for metal hydrogen systems. Z. Phys. Chem..

[32] Troyanov S.I., Snigireva E.M., Ling C.D. (2004). X-ray and neutron diffraction studies of Rb_4_LiH_3_(XO_4_)_4_ (X = S, Se) single crystals. Crystallogr. Rep..

[33] Belkova N.V., Shubina E.S., Epstein L.M. (2005). Diverse World of Unconventional Hydrogen Bonds. Acc. Chem. Res..

[34] Custelcean R., Jackson J. (2001). Dihydrogen Bonding: Structures, Energetics, and Dynamics. Chem. Rev..

[35] Crabtree R.H., Siegbahn P.E.M., Eisenstein O., Rheingold A.L., Koetzle T.F. (1996). A New Intermolecular Interaction: Unconventional Hydrogen Bonds with Element-Hydride Bonds as Proton Acceptor. Acc. Chem. Res..

[36] Grabowski S.J., Leszczynski J. (2011). Is a Dihydrogen Bond a Unique Phenomenon?. Computational Chemistry: Reviews of Current Trends.

[37] Bakhmutov V.I. (2007). Dihydrogen Bonds as Intermediates in Intermolecular Proton Transfer Reactions. Dihydrogen Bonds: Principles, Experiments, and Applications.

[38] Cramer C.J., Gladfelter W.L. (1997). Ab Initio Characterization of [H_3_N·BH_3_]_2_, [H_3_N·AlH_3_]_2_, and [H_3_N·GaH_3_]_2_. Inorg. Chem..

[39] Franken P.A., Hill A.E., Peters C.W., Weinreich G. (1961). Generation of Optical Harmonics. Phys. Rev. Lett..

[40] Boyd R.W. (2008). The Nonlinear Optical Susceptibility. Nonlinear Optics.

[41] Atuchin V.V., Kidyarov B.I., Pervukhina N.V. (2004). Phenomenological modeling and design of new acentric crystals for optoelectronics. Comput. Mater. Sci..

[42] Hedin L. (1965). New Method for Calculating the One-Particle Green’s Function with Application to the Electron-Gas Problem. Phys. Rev..

[43] Kresse G., Furthmüller J. (1996). Efficiency of ab-initio total energy calculations for metals and semiconductors using a plane-wave basis set. Comput. Mater. Sci..

[44] Kresse G., Furthmüller J. (1996). Efficient iterative schemes for Ab Initio Total-Energy Calc. Using A Plane-Wave Basis Set. Phys. Rev. B.

[45] Blöchl P.E. (1994). Projector augmented-wave method. Phys. Rev. B.

[46] Kresse G., Joubert D. (1999). From ultrasoft pseudopotentials to the projector augmented-wave method. Phys. Rev. B.

[47] Perdew J.P., Burke K., Ernzerhof M. (1996). Generalized Gradient Approximation Made Simple. Phys. Rev. Lett..

[48] Heyd J., Scuseria G.E., Ernzerhof M. (2003). Hybrid functionals based on a screened Coulomb potential. J. Chem. Phys..

[49] Krukau A.V., Vydrov O.A., Izmaylov A.F., Scuseria G.E. (2006). Influence of the exchange screening parameter on the performance of screened hybrid functionals. J. Chem. Phys..

[50] Henderson T.M., Paier J., Scuseria G.E. (2011). Accurate treatment of solids with the HSE screened hybrid. Phys. Status Solidi B.

[51] Moussa J.E., Schultz P.A., Chelikowsky J.R. (2012). Analysis of the Heyd-Scuseria-Ernzerhof density functional parameter space. J. Chem. Phys..

[52] Koller D., Blaha P., Tran F. (2013). Hybrid functionals for solids with an optimized Hartree-Fock mixing parameter. J. Phys. Condens. Matter.

[53] Marques M.A.L., Vidal J., Oliveira M.J.T., Reining L., Botti S. (2011). Density-based mixing parameter for hybrid functionals. Phys. Rev. B.

[54] He J., Franchini C. (2012). Screened hybrid functional applied to 3*d*^0^→3*d*^8^ transition-metal perovskites LaMO_3_ (M = Sc-Cu): Influence of the exchange mixing parameter on the structural, electronic, and magnetic properties. Phys. Rev. B.

[55] Gajdoš M., Hummer K., Kresse G., Furthmüller J., Bechstedt F. (2006). Linear optical properties in the projector-augmented wave methodology. Phys. Rev. B.

[56] Sharma S., Ambrosch-Draxl C. (2004). Second-Harmonic Optical Response from First Principles. Phys. Scr..

[57] The Elk FP-LAPW Code. http://elk.sourceforge.net/.

[58] Sanville E., Kenny S.D., Smith R., Henkelman G. (2007). Improved grid-based algorithm for Bader charge allocation. J. Comput. Chem..

[59] Tang W., Sanville E., Henkelman G. (2009). A grid-based Bader analysis algorithm without lattice bias. J. Phys. Condens. Matter.

[60] Bader R. (1994). Atoms in Molecules: A Quantum Theory.

[61] Savin A., Jepsen O., Flad J., Andersen O.K., Preuss H., von Schnering H.G. (1992). Electron Localization in Solid-State Structures of the Elements: The Diamond Structure. Angew. Chem. Int. Ed. Engl..

[62] Born M., Huang K. (1954). Dynamical Theory of Crystal Lattices.

[63] Ivantchev S., Kroumova E., Madariaga G., Pérez-Mato J.M., Aroyo M.I. (2000). *SUBGROUPGRAPH*: A Comput. Program Anal. Group- Relations Space Groups. J. Appl. Crystallogr..

[64] Kroumova E., Aroyo M.I., Pérez-Mato J.M., Kirov A., Capillas C., Ivantchev S., Wondratschek H. (2003). Bilbao Crystallographic Server I: Databases and crystallographic computing programs. Phase Trans..

[65] Bilbao Crystallographic Server. http://www.cryst.ehu.es/.

[66] Aroyo M., Pérez-Mato J., Orobengoa D., Tasci E., de la Flor G., Kirov A. (2011). Crystallography online: Bilbao Crystallographic Server. Bulg. Chem. Commun..

[67] Aroyo M., Pérez-Mato J., Capillas C., Kroumova E., Ivantchev S., Madariaga G., Kirov A., Wondratschek H. (2006). Bilbao Crystallographic Server I: Databases and crystallographic computing programs. Z. Krist..

[68] Aroyo M.I., Kirov A., Capillas C., Pérez-Mato J.M., Wondratschek H. (2006). Bilbao Crystallographic Server. II. Representations of crystallographic point groups and space groups. Acta Cryst..

[69] Stokes H.T., Hatch D.M., Campbell B.J. ISOTROPY Software Suite. http://stokes.byu.edu/iso/isotropy.php/.

[70] Stokes H.T., Hatch D.M. (2005). *FINDSYM*: Program Identifying Space-Group Symmetry A Crystal. J. Appl. Crystallogr..

[71] Momma K., Izumi F. (2011). *VESTA3* Three-Dimens. Vis. Crystal, Vol. Morphol. Data. J. Appl. Crystallogr..

[72] Pishtshev A., Karazhanov S.Z. (2014). Role of oxygen in materials properties of yttrium trihydride. Solid State Commun..

[73] Pishtshev A. (2017). Strong Effect of Anionic Boron-Induced Bonding in LiBSi_2_. Inorg. Chem..

[74] Pishtshev A., Rubin P. (2016). FeAs_2_ formation and electronic nematic ordering: Analysis in terms of structural transformations. Phys. Rev. B.

[75] Pishtshev A., Karazhanov S.Z. (2017). Structure-property relationships in cubic cuprous iodide: A novel view on stability, chemical bonding, and electronic properties. J. Chem. Phys..

[76] Bärnighausen H. (1980). Group-Subgroup Relations between Space Groups: A Useful Tool in Crystal Chemistry. MATCH Commun. Math. Chem..

[77] Köhler K.J. (1980). Subgroup-Relations between Crystallographic Groups. MATCH Commun. Math. Chem..

[78] Nye J.F. (1957). Physical Properties of Crystals. Their Representation by Tensor and Matrices.

[79] Gaillac R., Pullumbi P., Coudert F.X. (2016). ELATE: An open-source online application for analysis and visualization of elastic tensors. J. Phys. Condens. Matter..

[80] Gaillac R., Coudert F.X. ELATE: Elastic Tensor Analysis. http://progs.coudert.name/elate.

[81] Hill R. (1952). The Elastic Behaviour of a Crystalline Aggregate. Proc. Phys. Soc. Lond..

[82] Ranganathan S.I., Ostoja-Starzewski M. (2008). Universal Elastic Anisotropy Index. Phys. Rev. Lett..

[83] Kube C.M. (2016). Elastic anisotropy of crystals. AIP Adv..

[84] Chen X.Q., Niu H., Li D., Li Y. (2011). Modeling hardness of polycrystalline materials and bulk metallic glasses. Intermetallics.

[85] Tian Y., Xu B., Zhao Z. (2012). Microscopic theory of hardness and design of novel superhard crystals. Int. J. Refract. Met. Hard Mater..

