# Supplementary material for: On Prediction of a Novel Chiral Material Y2H3O(OH): A Hydroxyhydride Holding Hydridic and Protonic Hydrogens
Source: Materials (Basel). 2020 Feb 22;13(4):994. doi: 10.3390/ma13040994 (PMC7078701; doi:10.3390/ma13040994)
Supplement: Supplementary file 1 [file materials-13-00994-s001.pdf]

materials

Supplementary Materials

On Prediction of a Novel Chiral Material $Y_2H_3O(OH)$: A Hydroxyhydride Holding Hydridic and Protonic Hydrogens

Aleksandr Pishtshev [1], Evgenii Strugovshchikov [1,*] and Smagul Karazhanov [2]

[1] Institute of Physics, University of Tartu, W.Ostwaldi 1, 50411 Tartu, Estonia;
[2] Department for Solar Energy, Institute for Energy Technology, 2007 Kjeller, Norway
* Correspondence: evgenii.strugovshchikov@ut.ee

CONTENTS

1. Equilibrium atomic positions predicted for $M_2H_3O(OH)$ (M = Sc, La, Gd). Space group nr. 76 2

2. Structural stability of $M_2H_3O(OH)$ (M = Y, Sc, La, Gd) 3

2.1. Zone-centered vibrational modes calculated in the harmonic approximation 3

2.2. Eigenvalues (λ) of the stiffness matrix in GPa 8

2.3. Elastic properties of $M_2H_3O(OH)$ (M = Y, Sc, La, Gd) 9

3. Calculated X-ray diffraction patterns 11

4. Electronic properties 13

5. Pyroelectric properties 13

6. Optical properties 14

7. Evaluation of nonlinear optical properties 16

8. Vibrational data for $Y(OH)_3$ evaluated in the harmonic approximation 18

References 19

materials MDPI

1. Equilibrium Atomic Positions Predicted for $M_2H_3O(OH)$ (M = Sc, La, Gd). Space Group nr. 76

Table S1. $Sc_2H_3O(OH)$.

**$Sc_2H_3O(OH)$**				
**Atom**	**Wyckoff position**	**x**	**y**	**z**
Y (1)	4a	0.32233	0.20237	0.40469
Y (2)	4a	0.30878	0.20216	0.00863
O (1)	4a	0.43735	0.34728	0.20151
O (2)	4a	0.18382	0.32257	0.64637
H (1)	4a	0.01142	0.35204	0.06884
H (2)	4a	0.08719	0.45876	0.33599
H (3)	4a	0.10493	0.05170	0.19415
H (4)	4a	0.25790	0.19173	0.70484

Table S2. $La_2H_3O(OH)$.

**$La_2H_3O(OH)$**				
**Atom**	**Wyckoff position**	**x**	**y**	**z**
Y (1)	4a	0.30297	0.20478	0.40952
Y (2)	4a	0.30806	0.22846	0.98816
O (1)	4a	0.41205	0.36381	0.19960
O (2)	4a	0.19875	0.29784	0.67179
H (1)	4a	0.01545	0.40241	0.06714
H (2)	4a	0.06370	0.47004	0.33502
H (3)	4a	0.10928	0.07257	0.19322
H (4)	4a	0.28514	0.17917	0.70056

Table S3. $Gd_2H_3O(OH)$.

**$Gd_2H_3O(OH)$**				
**Atom**	**Wyckoff position**	**x**	**y**	**z**
Y (1)	4a	0.31261	0.20287	0.40738
Y (2)	4a	0.30815	0.21604	0.99757
O (1)	4a	0.42395	0.36050	0.19975
O (2)	4a	0.19129	0.31057	0.65676
H (1)	4a	0.01473	0.38205	0.06774
H (2)	4a	0.07369	0.46638	0.33789
H (3)	4a	0.10611	0.06439	0.19731
H (4)	4a	0.27302	0.18858	0.69804

materials

2. Structural Stability of $M_2H_3O(OH)$ (M = Y, Sc, La, Gd)

2.1. Zone-Centered Vibrational Modes Calculated in the Harmonic Approximation

Table S4. $Y_2H_3O(OH)$.

No	Frequency	Sublattice displacements
1	3336–3340 cm^{-1}	O(2)–H(4)
2	1393 cm^{-1}	H(1)–H(2)
3	1383 cm^{-1}	H(1)–H(2)–H(4)
4	1342 cm^{-1}	H(1)–H(2)
5	1241 cm^{-1}	H(2)
6	1239 cm^{-1}	H(2)–H(1)
7	1236 cm^{-1}	H(2)–H(1)
8	1107 cm^{-1}	H(2)–H(3)–H(1)–H(4)
9	1094 cm^{-1}	H(2)–H(1)–H(3)
10	1047 cm^{-1}	H(3)–H(1)–H(2)
11	1025 cm^{-1}	H(1)–H(2)–H(3)
12	1019 cm^{-1}	H(1)–H(2)–H(3)–H(4)
13	1008 cm^{-1}	H(2)–H(3)–H(1)–H(4)
14	933 cm−1	H(3)–H(4)–H(2)–H(1)–O(2)
15	930 cm^{-1}	H(3)–H(4)–H(2)–H(1)–O(2)
16	917 cm^{-1}	H(3)–H(4)–H(2)–H(1)–O(1)
17	915 cm^{-1}	H(3)–H(1)–H(4)–H(2)
18	900 cm^{-1}	H(3)–H(4)–H(2)–H(1)
19	882 cm^{-1}	H(2)–H(4)–H(1)–H(3)
20	881 cm^{-1}	H(2)–H(3)–H(4)–H(1)
21	880 cm^{-1}	H(3)–H(4)–H(1)–H(2)
22	856 cm^{-1}	H(1)–H(3)–H(2)–H(4)–O(1)
23	810 cm^{-1}	H(1)–H(2)–H(3)–H(4)–O(1)
24	787 cm^{-1}	H(3)–H(4)–H(1)–O(2)–O(1)
25	772 cm^{-1}	H(3)–H(1)–H(2)–H(4)
26	758 cm^{-1}	H(4)–H(3)–H(1)–H(2)–O(1)–O(2)
27	714 cm^{-1}	H(3)–H(4)–H(1)–H(2)–O(1)
28	679 cm^{-1}	H(4)–H(3)–H(1)–H(2)–O(1)–O(2)
29	622 cm^{-1}	H(4)–H(3)–H(1)–H(2)–O(1)–O(2)–Y(1)
30	586 cm^{-1}	O(1)–H(1)–H(4)–H(2)–H(3)–Y(2)
31	576 cm^{-1}	H(3)–H(2)–H(1)–O(2)
32	545 cm^{-1}	H(1)–H(4)–H(2)–O(2)–O(1)
33	527 cm^{-1}	O(1)–Y(2)–O(2)–Y(1)–H(4)–H(3)
34	517 cm^{-1}	O(1)–O(2)–H(4)–H(1)–H(2)–H(3)–Y(2)
35	421 cm^{-1}	O(1)–Y(2)–H(4)–H(2)

36	392 cm^{-1}	O(1)–O(2)–Y(1)–H(3)–H(4)
37	371 cm^{-1}	O(1)–O(2)–Y(1)–Y(2)–H(4)–H(1)
38	367 cm^{-1}	O(1)–O(2)–Y(1)–Y(2)–H(3)
39	333 cm^{-1}	O(1)–Y(2)–Y(1)–H(2)
40	325 cm^{-1}	O(2)–Y(1)–Y(2)–H(4)
41	322 cm^{-1}	O(2)–O(1)–Y(1)–Y(2)–H(4)
42	317 cm^{-1}	O(2)–H(4)–Y(1)–Y(2)–O(1)–H(1)
43	312 cm^{-1}	O(1)–O(2)–Y(1)–Y(2)–H(4)
44	300 cm^{-1}	O(2)–O(1)–Y(2)–Y(1)–H(4)
45	280 cm^{-1}	O(2)–Y(1)–Y(2)–O(1)–H(4)
46	263 cm^{-1}	O(2)–O(1)–Y(1)–Y(2)–H(3)–H(4)
47	258 cm^{-1}	O(1)–O(2)–Y(1)–Y(2)–H(3)–H(4)
48	234 cm^{-1}	Y(1)–Y(2)–O(1)–O(2)–H(4)
49	232 cm^{-1}	Y(1)–Y(2)–O(1)–O(2)
50	226 cm^{-1}	Y(1)–Y(2)–O(1)–O(2)
51	216 cm^{-1}	Y(1)–Y(2)–O(1)–O(2)–H(4)
52	215 cm^{-1}	Y(2)–O(2)–O(1)–H(2)
53	210 cm^{-1}	Y(1)–Y(2)–O(1)–O(2)
54	179–181 cm^{-1}	Y(1)–Y(2)–O(1)–O(2)
55	169 cm^{-1}	Y(1)–Y(2)–O(2)
56	116–157 cm^{-1}	Y(1)–Y(2)–O(1)–O(2)
57	106–115 cm^{-1}	Y(2)–Y(1)–O(2)–O(1)
58	79–94 cm^{-1}	Y(1)–Y(2)–O(1)–O(2)

Table S5. $Sc_2H_3O(OH)$.

No	Frequency	Sublattice displacements
1	3242–3245 cm^{-1}	O(2)–H(4)
2	1469 cm^{-1}	H(1)–H(2)
3	1457 cm^{-1}	H(1)–H(2)–H(4)
4	1413 cm^{-1}	H(1)–H(2)
5	1352 cm^{-1}	H(2)
6	1346 cm^{-1}	H(2)–H(1)
7	1341 cm^{-1}	H(2)–H(1)
8	1150 cm^{-1}	H(2)–H(3)–H(1)–H(4)
9	1132 cm^{-1}	H(2)–H(1)–H(3)
10	1107 cm^{-1}	H(3)–H(1)–H(2)
11	1079 cm^{-1}	H(1)–H(2)–H(3)
12	1078 cm^{-1}	H(1)–H(2)–H(3)–H(4)
13	1050 cm^{-1}	H(2)–H(3)–H(1)–H(4)
14	993 cm^{-1}	H(3)–H(4)–H(2)–H(1)–O(2)
15	983 cm^{-1}	H(3)–H(4)–H(2)–H(1)–O(2)
16	968 cm^{-1}	H(3)–H(4)–H(2)–H(1)–O(1)

17	957 cm^{-1}	H(3)–H(1)–H(4)–H(2)
18	953 cm^{-1}	H(3)–H(4)–H(2)–H(1)
19	933 cm^{-1}	H(2)–H(4)–H(1)–H(3)
20	921 cm^{-1}	H(2)–H(3)–H(4)–H(1)
21	918 cm^{-1}	H(3)–H(4)–H(1)–H(2)
22	815 cm^{-1}	H(1)–H(3)–H(2)–H(4)–O(1)
23	887 cm^{-1}	H(1)–H(2)–H(3)–H(4)–O(1)
24	840 cm^{-1}	H(3)–H(4)–H(1)–O(2)–O(1)
25	823 cm^{-1}	H(3)–H(1)–H(2)–H(4)
26	820 cm^{-1}	H(4)–H(3)–H(1)–H(2)–O(1)–O(2)
27	782 cm^{-1}	H(3)–H(4)–H(1)–H(2)–O(1)
28	757–761 cm^{-1}	H(4)–H(3)–H(1)–H(2)–O(1)–O(2)
29	733 cm^{-1}	H(4)–H(3)–H(1)–H(2)–O(1)–O(2)–Sc(1)
30	667 cm^{-1}	O(1)–H(1)–H(4)–H(2)–H(3)–Sc(2)
31	655 cm^{-1}	H(3)–H(2)–H(1)–O(2)
32	608 cm^{-1}	H(1)–H(4)–H(2)–O(2)–O(1)
33	584 cm^{-1}	O(1)–Y(2)–O(2)–Sc(1)–H(4)–H(3)
34	574 cm^{-1}	O(1)–O(2)–H(4)–H(1)–H(2)–H(3)–Sc(2)
35	501 cm^{-1}	O(1)–Sc(2)–H(4)–H(2)
36	456 cm^{-1}	O(1)–O(2)–Sc(1)–H(3)–H(4)
37	424 cm^{-1}	O(1)–O(2)–Sc(1)–Sc(2)–H(4)–H(1)
38	423 cm^{-1}	O(1)–O(2)–Sc(1)–Sc(2)–H(3)
39	411 cm^{-1}	O(1)–Sc(2)–Sc(1)–H(2)
40	385 cm^{-1}	O(2)–Sc(1)–Sc(2)–H(4)
41	377 cm^{-1}	O(2)–O(1)–Sc(1)–Sc(2)–H(4)
42	368 cm^{-1}	O(2)–H(4)–Sc(1)–Sc(2)–O(1)–H(1)
43	366 cm^{-1}	O(1)–O(2)–Sc(1)–Sc(2)–H(4)
44	351 cm^{-1}	O(2)–O(1)–Sc(2)–Sc(1)–H(4)
45	344 cm^{-1}	O(2)–Sc(1)–Sc(2)–O(1)–H(4)
46	295–310 cm^{-1}	O(2)–O(1)–Sc(1)–Sc(2)–H(3)–H(4)
47	267—287 cm^{-1}	O(1)–O(2)–Sc(1)–Sc(2)–H(3)–H(4)
48	265 cm^{-1}	Sc(1)–Sc(2)–O(1)–O(2)–H(4)
49	264 cm^{-1}	Sc(1)–Sc(2)–O(1)–O(2)
50	250 cm^{-1}	Sc(1)–Sc(2)–O(1)–O(2)
51	239 cm^{-1}	Sc(1)–Sc(2)–O(1)–O(2)–H(4)
52	224 cm^{-1}	Sc(2)–O(2)–O(1)–H(2)
53	206 cm^{-1}	Sc(1)–Sc(2)–O(1)–O(2)
54	190–100 cm^{-1}	Sc(1)–Sc(2)–O(1)–O(2)
55	169 cm^{-1}	Sc(1)–Sc(2)–O(2)
56	150–153 cm^{-1}	Sc(1)–Sc(2)–O(1)–O(2)
57	117–135 cm^{-1}	Sc(2)–Sc(1)–O(2)–O(1)
58	91 cm^{-1}	Sc(1)–Sc(2)–O(1)–O(2)

Table S6. $La_2H_3O(OH)$.

No	Frequency	Sublattice displacements
1	3347–3348 cm^{-1}	O(2)–H(4)
2	1244 cm^{-1}	H(1)–H(2)
3	1224 cm^{-1}	H(1)–H(2)–H(4)
4	1159 cm^{-1}	H(1)–H(2)
5	1074 cm^{-1}	H(2)
6	1068 cm^{-1}	H(2)–H(1)
7	1055 cm^{-1}	H(2)–H(1)
8	1022 cm^{-1}	H(2)–H(3)–H(1)–H(4)
9	999 cm^{-1}	H(2)–H(1)–H(3)
10	945 cm^{-1}	H(3)–H(1)–H(2)
11	927 cm^{-1}	H(1)–H(2)–H(3)
12	914 cm^{-1}	H(1)–H(2)–H(3)–H(4)
13	901 cm^{-1}	H(2)–H(3)–H(1)–H(4)
14	877 cm^{-1}	H(3)–H(4)–H(2)–H(1)–O(2)
15	869 cm^{-1}	H(3)–H(4)–H(2)–H(1)–O(2)
16	866 cm^{-1}	H(3)–H(4)–H(2)–H(1)–O(1)
17	846 cm^{-1}	H(3)–H(1)–H(4)–H(2)
18	843 cm^{-1}	H(3)–H(4)–H(2)–H(1)
19	833 cm^{-1}	H(2)–H(4)–H(1)–H(3)
20	818 cm^{-1}	H(2)–H(3)–H(4)–H(1)
21	806–813 cm^{-1}	H(3)–H(4)–H(1)–H(2)
22	792 cm^{-1}	H(1)–H(3)–H(2)–H(4)–O(1)
23	763 cm^{-1}	H(1)–H(2)–H(3)–H(4)–O(1)
24	695 cm^{-1}	H(3)–H(4)–H(1)–O(2)–O(1)
25	694 cm^{-1}	H(3)–H(1)–H(2)–H(4)
26	674 cm^{-1}	H(4)–H(3)–H(1)–H(2)–O(1)–O(2)
27	640 cm^{-1}	H(3)–H(4)–H(1)–H(2)–O(1)
28	611 cm^{-1}	H(4)–H(3)–H(1)–H(2)–O(1)–O(2)
29	603 cm^{-1}	H(4)–H(3)–H(1)–H(2)–O(1)–O(2)–La(1)
30	602 cm^{-1}	O(1)–H(1)–H(4)–H(2)–H(3)–La(2)
31	596 cm^{-1}	H(3)–H(2)–H(1)–O(2)
32	586 cm^{-1}	H(1)–H(4)–H(2)–O(2)–O(1)
33	519 cm^{-1}	O(1)–La(2)–O(2)–Y(1)–H(4)–H(3)
34	469 cm^{-1}	O(1)–O(2)–H(4)–H(1)–H(2)–H(3)–La(2)
35	428 cm^{-1}	O(1)–La(2)–H(4)–H(2)
36	412 cm^{-1}	O(1)–O(2)–La(1)–H(3)–H(4)
37	388 cm^{-1}	O(1)–O(2)–La(1)–La(2)–H(4)–H(1)
38	387 cm^{-1}	O(1)–O(2)–La(1)–La(2)–H(3)
39	337 cm^{-1}	O(1)–La(2)–La(1)–H(2)
40	316 cm^{-1}	O(2)–La(1)–La(2)–H(4)

41	304 cm^{-1}	O(2)–O(1)–La(1)–La(2)–H(4)
42	303 cm^{-1}	O(2)–H(4)–La(1)–La(2)–O(1)–H(1)
43	302 cm^{-1}	O(1)–O(2)–La(1)–La(2)–H(4)
44	279 cm^{-1}	O(2)–O(1)–La(2)–La(1)–H(4)
45	266 cm^{-1}	O(2)–La(1)–La(2)–O(1)–H(4)
46	243–255 cm^{-1}	O(2)–O(1)–La(1)–La(2)–H(3)–H(4)
47	200–211 cm^{-1}	O(1)–O(2)–La(1)–La(2)–H(3)–H(4)
48	188 cm^{-1}	La(1)–La(2)–O(1)–O(2)–H(4)
49	181 cm^{-1}	La(1)–La(2)–O(1)–O(2)
50	178 cm^{-1}	La(1)–La(2)–O(1)–O(2)
51	168 cm^{-1}	La(1)–La(2)–O(1)–O(2)–H(4)
52	167 cm^{-1}	La(2)–O(2)–O(1)–H(2)
53	162 cm^{-1}	La(1)–La(2)–O(1)–O(2)
54	139–143 cm^{-1}	La(1)–La(2)–O(1)–O(2)
55	126 cm^{-1}	La(1)–La(2)–O(2)
56	106–116 cm^{-1}	La(1)–La(2)–O(1)–O(2)
57	96–101 cm^{-1}	La(2)–La(1)–O(2)–O(1)
58	65–86 cm^{-1}	La(1)–La(2)–O(1)–O(2)

Table S7. $Gd_2H_3O(OH)$.

No	Frequency	Sublattice displacements
1	3343–3344 cm^{-1}	O(2)–H(4)
2	1390 cm^{-1}	H(1)–H(2)
3	1377 cm^{-1}	H(1)–H(2)–H(4)
4	1333 cm^{-1}	H(1)–H(2)
5	1212 cm^{-1}	H(2)
6	1212 cm^{-1}	H(2)–H(1)
7	1208 cm^{-1}	H(2)–H(1)
8	1100 cm^{-1}	H(2)–H(3)–H(1)–H(4)
9	1089 cm^{-1}	H(2)–H(1)–H(3)
10	1043 cm^{-1}	H(3)–H(1)–H(2)
11	1020 cm^{-1}	H(1)–H(2)–H(3)
12	1005 cm^{-1}	H(1)–H(2)–H(3)–H(4)
13	987 cm^{-1}	H(2)–H(3)–H(1)–H(4)
14	930 cm^{-1}	H(3)–H(4)–H(2)–H(1)–O(2)
15	928 cm^{-1}	H(3)–H(4)–H(2)–H(1)–O(2)
16	916 cm^{-1}	H(3)–H(4)–H(2)–H(1)–O(1)
17	913 cm^{-1}	H(3)–H(1)–H(4)–H(2)
18	904 cm^{-1}	H(3)–H(4)–H(2)–H(1)
19	893 cm^{-1}	H(2)–H(4)–H(1)–H(3)
20	877 cm^{-1}	H(2)–H(3)–H(4)–H(1)
21	874 cm^{-1}	H(3)–H(4)–H(1)–H(2)

22	862 cm^{-1}	H(1)–H(3)–H(2)–H(4)–O(1)
23	821 cm^{-1}	H(1)–H(2)–H(3)–H(4)–O(1)
24	776 cm^{-1}	H(3)–H(4)–H(1)–O(2)–O(1)
25	768 cm^{-1}	H(3)–H(1)–H(2)–H(4)
26	753 cm^{-1}	H(4)–H(3)–H(1)–H(2)–O(1)–O(2)
27	704 cm^{-1}	H(3)–H(4)–H(1)–H(2)–O(1)
28	702 cm^{-1}	H(3)–H(4)–H(1)–H(2)–O(1)
29	667 cm^{-1}	H(4)–H(3)–H(1)–H(2)–O(1)–O(2)
30	612 cm^{-1}	H(4)–H(3)–H(1)–H(2)–O(1)–O(2)–Gd(1)
31	606 cm^{-1}	O(1)–H(1)–H(4)–H(2)–H(3)–Gd(2)
32	571 cm^{-1}	H(3)–H(2)–H(1)–O(2)
33	534 cm^{-1}	H(1)–H(4)–H(2)–O(2)–O(1)
34	519 cm^{-1}	O(1)–Gd(2)–O(2)–Gd(1)–H(4)–H(3)
35	500 cm^{-1}	O(1)–O(2)–H(4)–H(1)–H(2)–H(3)–Gd(2)
36	414 cm^{-1}	O(1)–Gd(2)–H(4)–H(2)
37	383 cm^{-1}	O(1)–O(2)–Gd(1)–H(3)–H(4)
38	365 cm^{-1}	O(1)–O(2)–Gd(1)–Gd(2)–H(4)–H(1)
39	361 cm^{-1}	O(1)–O(2)–Gd(1)–Gd(2)–H(3)
40	325 cm^{-1}	O(1)–Gd(2)–Gd(1)–H(2)
41	317 cm^{-1}	O(2)–Gd(1)–Gd(2)–H(4)
42	317 cm^{-1}	O(2)–O(1)–Gd(1)–Gd(2)–H(4)
43	313 cm^{-1}	O(2)–H(4)–Gd(1)–Gd(2)–O(1)–H(1)
44	303 cm^{-1}	O(1)–O(2)–Gd(1)–Gd(2)–H(4)
45	297 cm^{-1}	O(2)–O(1)–Gd(2)–Gd(1)–H(4)
46	272 cm^{-1}	O(2)–Gd(1)–Gd(2)–O(1)–H(4)
47	213 cm^{-1}	O(2)–Gd(1)–Gd(2)–O(1)–H(4)
48	206 cm^{-1}	O(2)–O(1)–Gd(1)–Gd(2)–H(3)–H(4)
49	202 cm^{-1}	O(1)–O(2)–Gd(1)–Gd(2)–H(3)–H(4)
50	184 cm^{-1}	Gd(1)–Gd(2)–O(1)–O(2)–H(4)
51	178 cm^{-1}	Gd(1)–Gd(2)–O(1)–O(2)
52	172 cm^{-1}	Gd(1)–Gd(2)–O(1)–O(2)
53	141 cm^{-1}	Gd(1)–Gd(2)–O(1)–O(2)–H(4)
54	139 cm^{-1}	Gd(2)–O(2)–O(1)–H(2)
55	132 cm^{-1}	Gd(1)–Gd(2)–O(1)–O(2)
56	107–125 cm^{-1}	Gd(1)–Gd(2)–O(1)–O(2)
57	101 cm^{-1}	Gd(1)–Gd(2)–O(2)
58	93–97 cm^{-1}	Gd(1)–Gd(2)–O(1)–O(2)
59	84–92 cm^{-1}	Gd(2)–Gd(1)–O(2)–O(1)
60	72–82 cm^{-1}	Gd(1)–Gd(2)–O(1)–O(2)

2.2. Eigenvalues (λ) of the Stiffness Matrix in GPa

Table S8. $M_2H_3O(OH)$ (M = Y, Sc, La, Gd).

-	λ_1, GPa	λ_2, GPa	λ_3, GPa	λ_4, GPa	λ_5, GPa	λ_6, GPa
$Y_2H_3O(OH)$	41.0	50.8	50.8	81.0	93.6	194.9
$Sc_2H_3O(OH)$	45.2	54.9	54.9	88.3	111.6	239.7
$La_2H_3O(OH)$	26.7	36.1	36.1	52.9	65.4	124.0
$Gd_2H_3O(OH)$	39.8	49.2	49.2	80.1	89.4	189.5

2.3. Elastic Properties of $M_2H_3O(OH)$ (M = Y, Sc, La, Gd)

Table S9. Nonzero components of the elasticity for $M_2H_3O(OH)$ (M = Sc, La, Gd) in GPa.

-	C_{11}	C_{12}	C_{13}	C_{33}	C_{16}	C_{44}	C_{66}
$Sc_2H_3O(OH)$	140.2	60.8	41.6	150.4	12.3	54.9	54.1
$La_2H_3O(OH)$	79.6	28.2	18.5	81.6	4.3	36.1	28.2
$Gd_2H_3O(OH)$	121.5	44.3	30.1	113.2	7.3	49.2	42.6

Table 10. Summary of aggregate parameters calculated for $M_2H_3O(OH)$ (M = Sc, La, Gd).

-	B, GPa	E, GPa	G, GPa	G/B	ν	γ	θ_D, K	H_V
$Sc_2H_3O(OH)$	79.8	125.4	50.6	0.63	0.24	1.45	658	8.7/8.8
$La_2H_3O(OH)$	41.2	74.9	31.3	0.76	0.20	1.29	347	7.9/7.7
$Gd_2H_3O(OH)$	62.6	107.8	44.5	0.71	0.21	1.32	383	9.4/9.2

Table S11. Fulfilment of the Born stability conditions [1] for the tetragonal phase of $M_2H_3O(OH)$ (M = Y, Sc, La, Gd).

-	Born stability conditions	$Y_2H_3O(OH)$	$Sc_2H_3O(OH)$	$La_2H_3O(OH)$	$Gd_2H_3O(OH)$
1	$C_{11} > \lvert C_{12} \rvert$	123.3 > 45.2	140.2 > 60.8	79.6 > 28.2	121.5 > 44.3
2	$2C_{13}^2 < C_{33}(C_{11} + C_{12})$	1984.5 < 20220	3461.1 < 30230	684.5 < 8796.5	1812 < 18768.6
3	$C_{44} > 0$	50.8 > 0	54.9 > 0	36.1 > 0	49.2 > 0
4	$2C_{16}^2 < C_{66}(C_{11}–C_{12})$	106.6 < 3428.6	302.6 < 4295.5	37 < 1449.5	106.6 < 3288.7

The elastic moduli have been evaluated within the Voigt-Reuss-Hill averaging approach [2]. To quantify the anisotropy of the elastic behavior of $M_2H_3O(OH)$ (M = Y, Sc, La, Gd) the relative (the universal anisotropy index A^U) and absolute (A^L) measures of anisotropy have been estimated according to relations given in [3,4], respectively. Both indexes are expressed in terms of the Voigt [5] and Reuss [6] bounds on the bulk and shear modulus as follows:

$$A^U = \frac{B^V}{B^R} + 5\frac{G^V}{G^R} - 6, \quad A^L = \sqrt{\left[ln\left(\frac{B^V}{B^R}\right)\right]^2 + 5\left[ln\left(\frac{G^V}{G^R}\right)\right]^2}.$$

Since for the completely isotropic body $A^U = A^L \equiv 0$, nonzero values of the indexes A^U and A^L determine the magnitude of the elastic anisotropy. The longitudinal elastic anisotropy for the tetragonal body was estimated via the relation $C_{33} = C_{11}$. The interplay of elastic and plastic properties underlies the hardness of a system. This characteristic has been evaluated in terms of the Vickers hardness, H_V, by using two semi-empirical model relations proposed in [7,8].

Quantity	Eq. of Ref.[6]	Eq. of Ref.[7]
H_V	$= 2\,[(G/B)^2\,G]^{0.585} - 3$	$= 0.92\,(G/B)^{1.137}\,G^{0.708}$

For the tetragonal system the Cauchy pressure in the plane of lateral shear is determined from the difference [9] C_{12}-C_{66}.

For the polycrystalline material the mean value of the acoustic wave velocity, v_m, averaged via the longitudinal ($v_\parallel$) and shear ($v_\perp$) elastic wave velocities, may be expressed in terms of the elastic moduli and the density of the material [10]:

$$v_m = \left[\frac{1}{3}\left(\frac{1}{v_\parallel^3} + \frac{2}{v_\perp^3}\right)\right]^{-1/3}, \quad v_\parallel = \left[\left(B + \frac{4}{3}G\right)/\rho\right]^{1/2}, \quad v_\perp = \left(\frac{G}{\rho}\right)^{1/2}.$$

In the Anderson version [10] of Debye-type phonon model the mean sound velocity determines the Debye temperature (θ_D):

$$k_B\Theta_D = hv_m\left[\frac{3n}{4\pi}\left(\frac{N_A\rho}{M}\right)\right]^{1/3}.$$

In the similar semi-empirical context of the Debye approximation, suggested and verified in [11], one can assess the Grüneisen parameter (γ) numerically as:

$$\gamma = \frac{3}{2}\left(\frac{1+\mu}{2-3\mu}\right).$$

3. Calculated X-Ray Diffraction Patterns

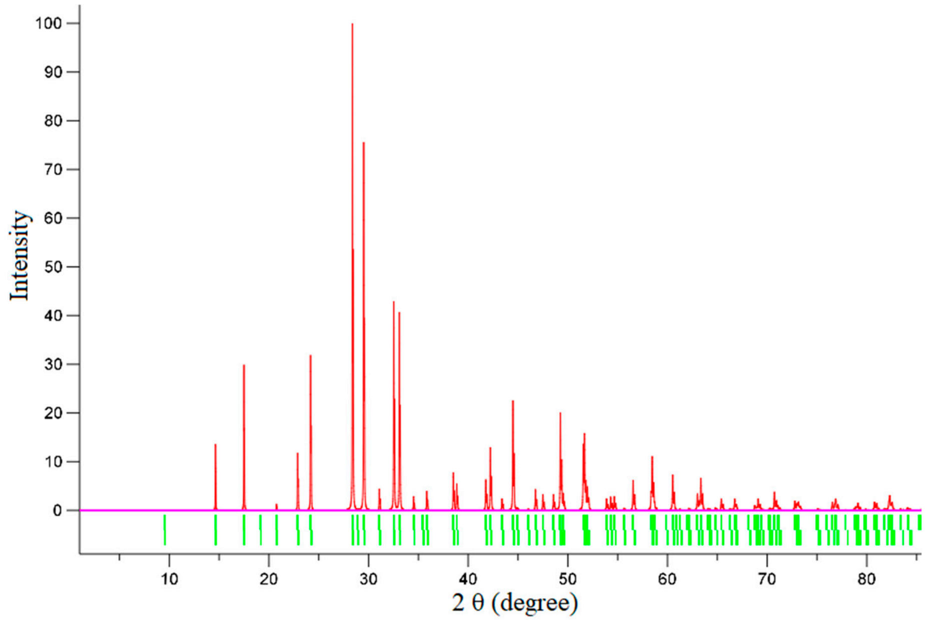

Figure S1: X-ray diffraction pattern for the $Y_2H_3O(OH)$ structure.

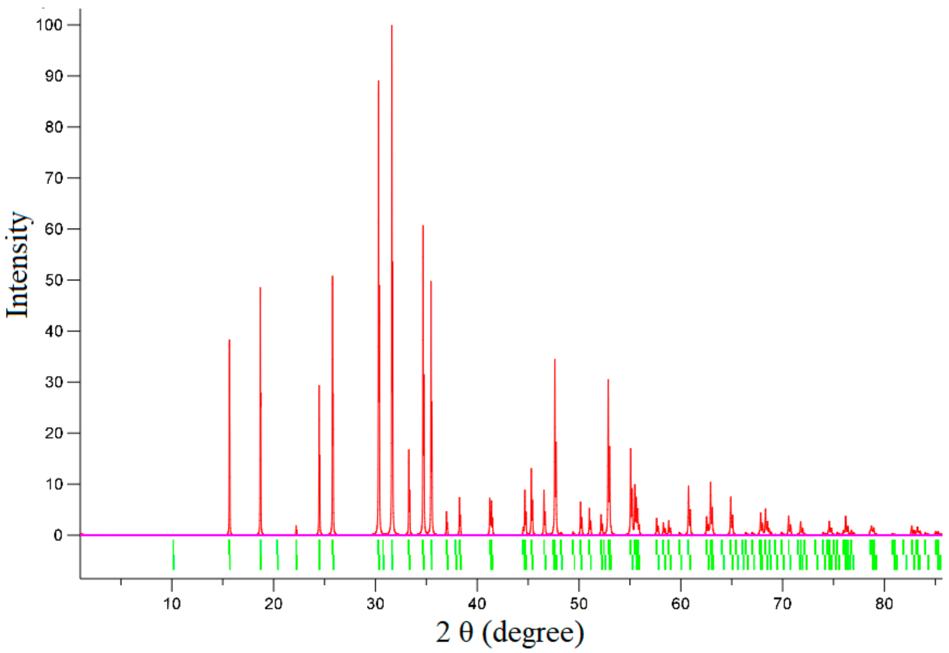

Figure S2: X-ray diffraction pattern for the $Sc_2H_3O(OH)$ structure.

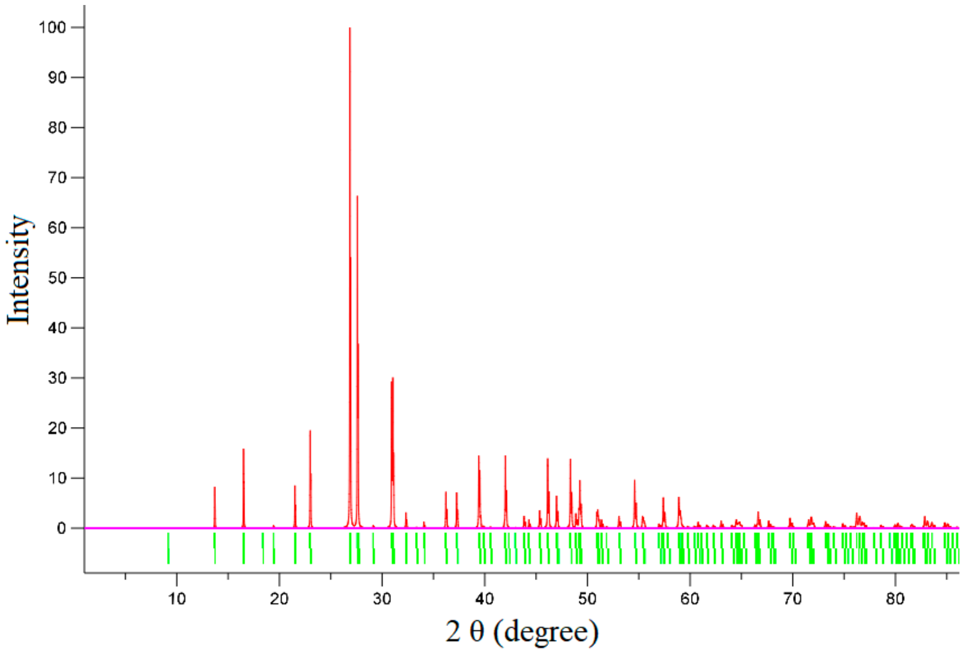

Figure S3: X-ray diffraction pattern for the $La_2H_3O(OH)$ structure.

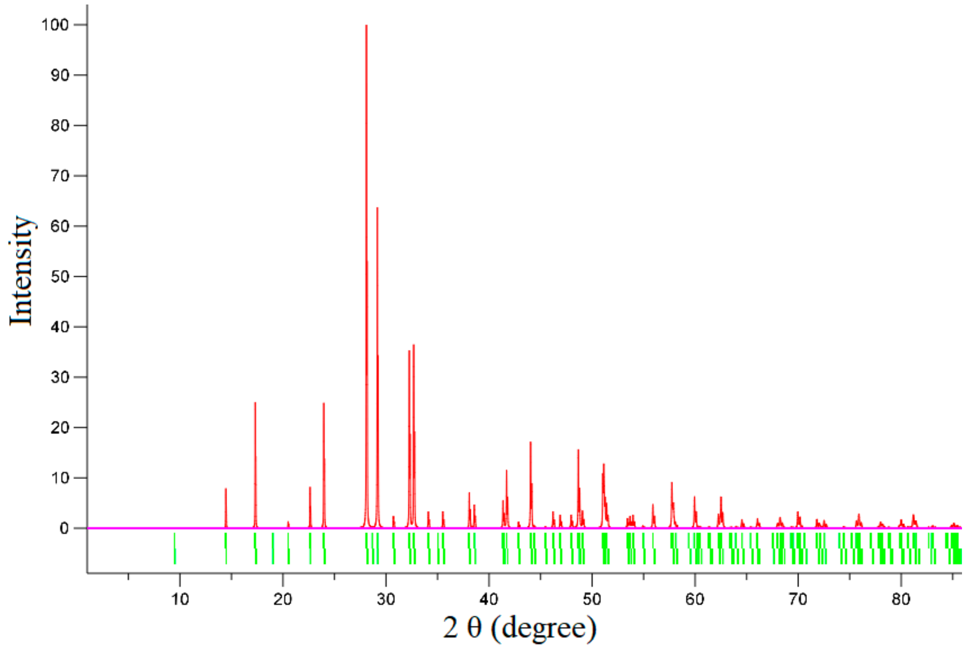

Figure S4. X-ray diffraction pattern for the $Gd_2H_3O(OH)$ structure.

4. Electronic Properties

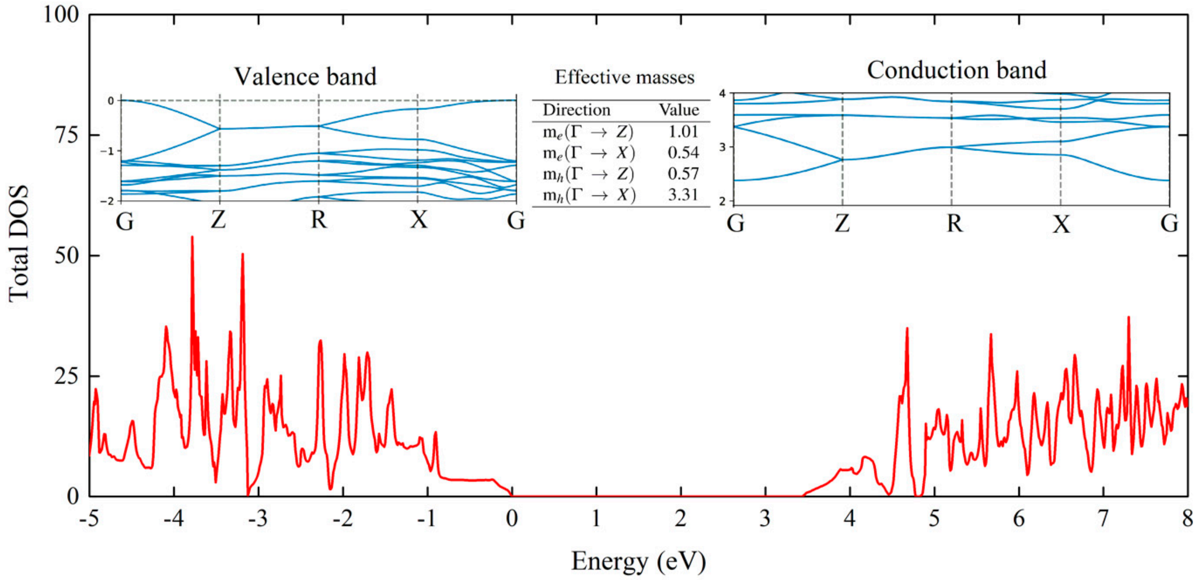

Figure S5. The total density of states, band structure and effective masses of $Y_2H_3O(OH)$. The total DOS was calculated by using the HSE-06 hybrid functional. Fundamental band gap is 3.4 eV.

5. Pyroelectric Properties

Nonvanishing component of the electric polarization vector for the $P4_1$ tetragonal structure of $Y_2H_3O(OH)$ is P_z. The evaluation of P_z, which was performed by using computational procedures implemented in VASP, allowed us to predict a value of about 0.87 C/m^2.

materials

6. Optical Properties

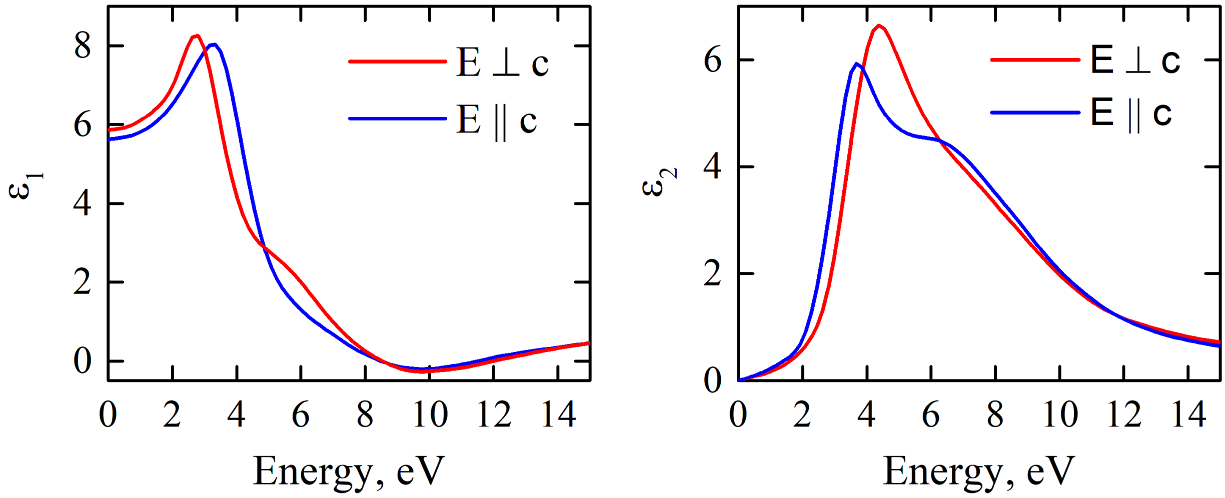

Figure S6: Real and imaginary part of dielectric function of the $Y_2H_3O(OH)$. The dielectric function was calculated by using the G_0W_0 approximation.

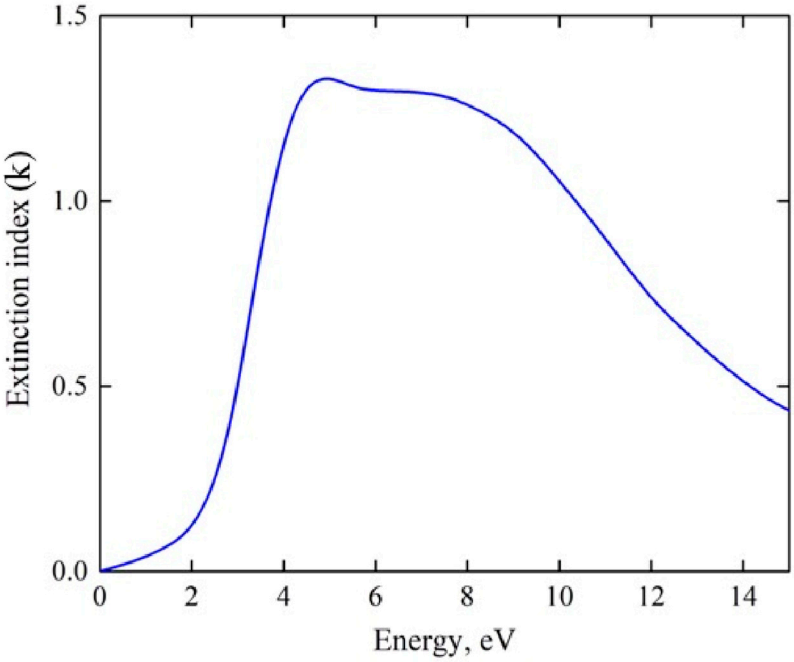

Figure S7: Spectral behavior of extinction index in $Y_2H_3O(OH)$.

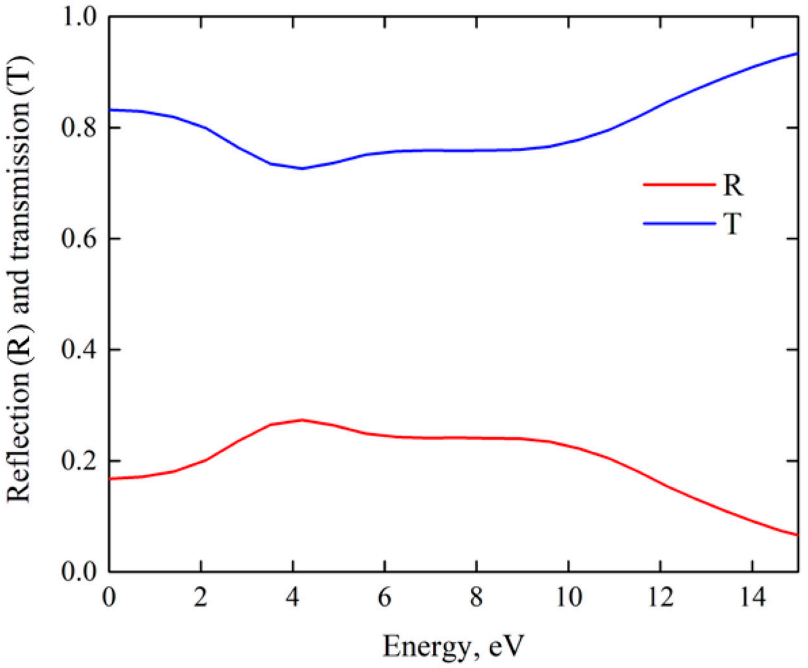

Figure S8: Spectral behavior of reflection (R) and transmission (T) in $Y_2H_3O(OH)$.

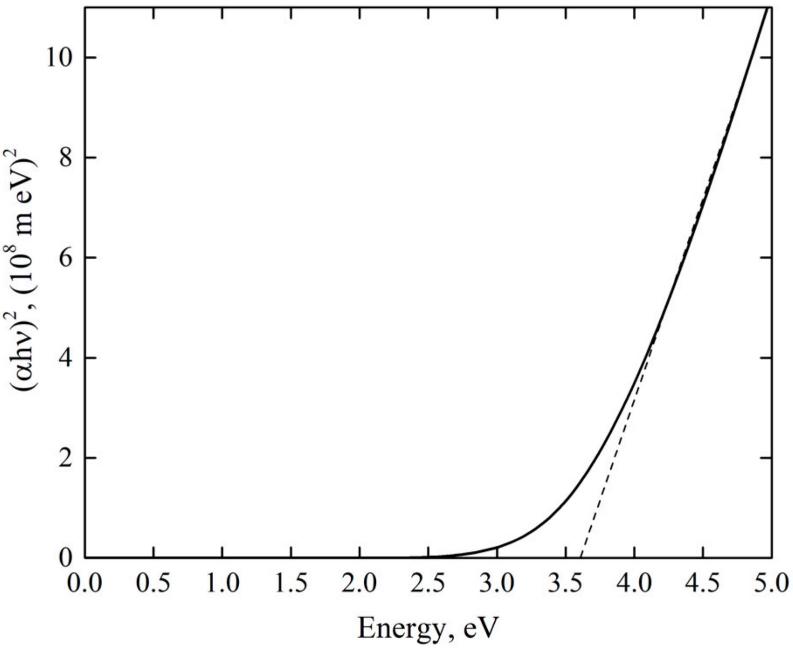

Figure S9: The Tauc plot for $Y_2H_3O(OH)$. The evaluated value of the optical band gap is 3.6 eV.

7. Evaluation of Nonlinear Optical Properties

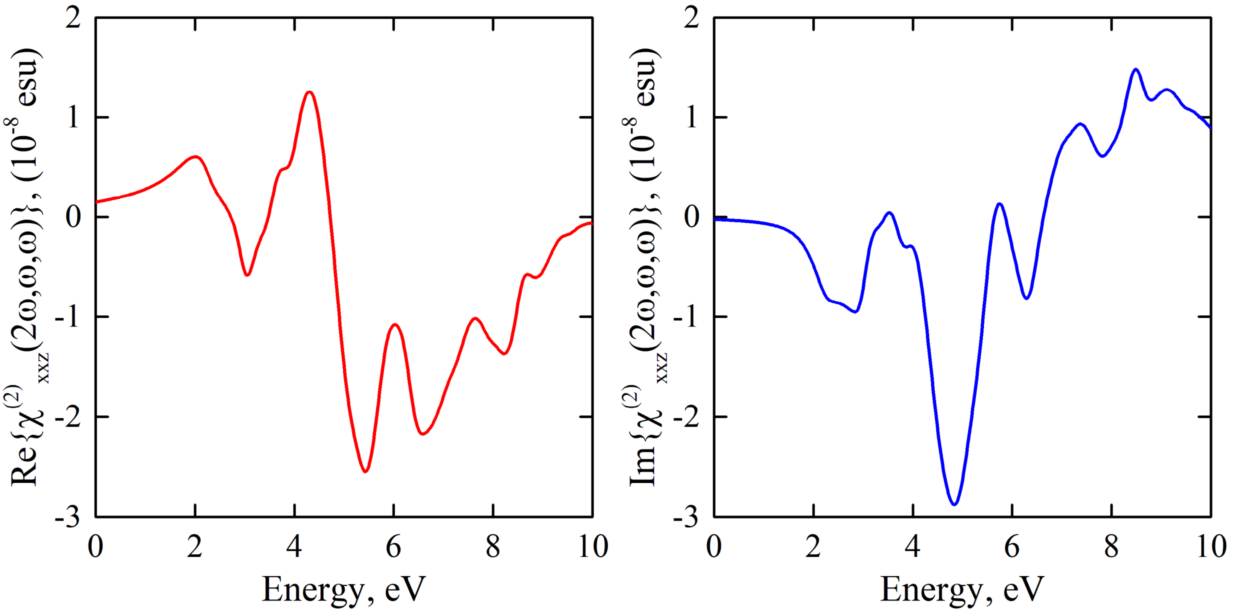

Figure S10. Second-harmonic generation (SHG) spectrum of $Y_2H_3O(OH)$ represented in terms of the spectral behavior of tensor component $\chi_{xxz}(2\omega, \omega, \omega)$.

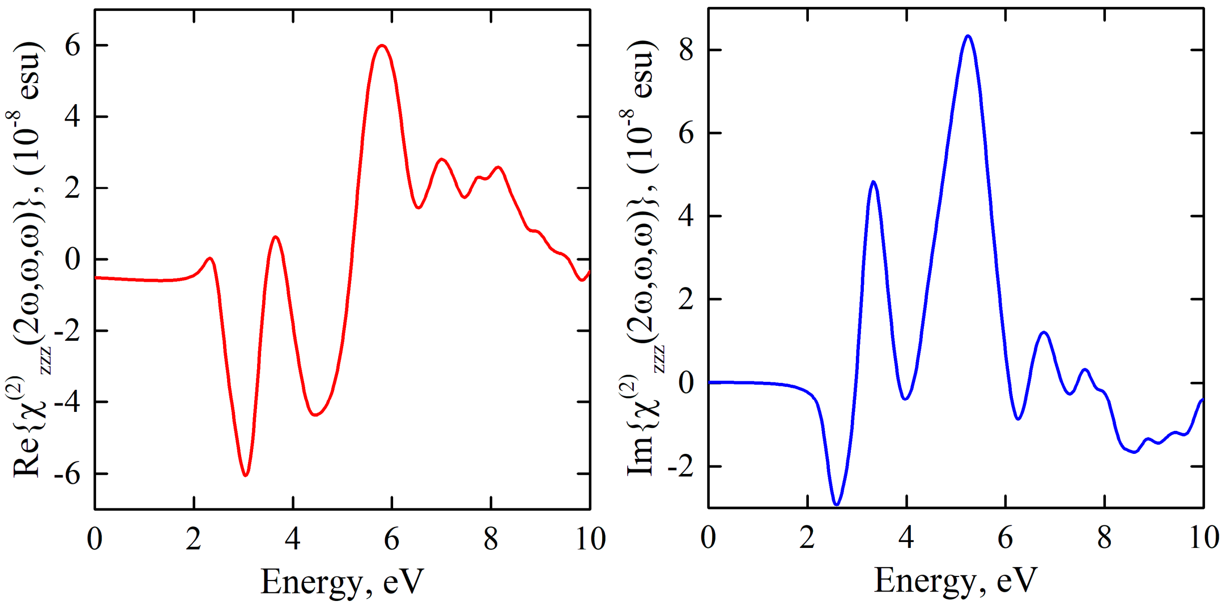

Figure S11: Second-harmonic generation (SHG) spectrum of $Y_2H_3O(OH)$ represented in terms of the spectral behavior of tensor component $\chi_{zzz}(2\omega, \omega, \omega)$.

Materials **2020**, *13*, 994; doi: 10.3390/ma13040994 www.mdpi.com/journal/materials

materials MDPI

Table S12. Components of the second-order susceptibility tensor of $Y_2H_3O(OH)$ evaluated for different photon wavelengths.

		λ = 1064 nm		
Index	Re{$\chi^{(2)}$(2ω,ω,ω)}, pm/V	Im{ $\chi^{(2)}$(2ω,ω,ω)}, pm/V	∣ $\chi^{(2)}$(2ω,ω,ω)∣, pm/V	n
xxz	1.332	–0.327	1.372	2.098
zzz	–2.463	–0.047	3.226	2.464
		λ = 460 nm		
Index	Re{$\chi^{(2)}$(2ω,ω,ω)}, pm/V	Im{ $\chi^{(2)}$(2ω,ω,ω)}, pm/V	∣ $\chi^{(2)}$(2ω,ω,ω)∣, pm/V	n
xxz	0.141	–3.816	3.818	2.185
zzz	–13.751	–11.046	17.638	2.217
		λ = 422 nm		
Index	Re{$\chi^{(2)}$(2ω,ω,ω)}, pm/V	Im{ $\chi^{(2)}$(2ω,ω,ω)}, pm/V	∣ $\chi^{(2)}$(2ω,ω,ω)∣, pm/V	n
xxz	–1.778	–3.620	4.033	2.209
zzz	–23.467	–2.371	23.586	2.244
		λ = 400 nm		
Index	Re{$\chi^{(2)}$(2ω,ω,ω)}, pm/V	Im{ $\chi^{(2)}$(2ω,ω,ω)}, pm/V	∣ $\chi^{(2)}$(2ω,ω,ω)∣, pm/V	n
xxz	–2.314	–1.595	2.811	2.227
zzz	–24.699	9.449	26.445	2.265
		λ = 397.5 nm		
Index	Re{$\chi^{(2)}$(2ω,ω,ω)}, pm/V	Im{ $\chi^{(2)}$(2ω,ω,ω)}, pm/V	∣ $\chi^{(2)}$(2ω,ω,ω)∣, pm/V	n
xxz	–2.206	–1.366	2.595	2.229
zzz	–24.129	11.039	26.534	2.268

8. Vibrational Data for $Y(OH)_3$ Evaluated in the Harmonic Approximation

Table S13. $Y(OH)_3$.

No	Frequency	Sublattice displacements
1	3683–3696 cm^{-1}	O–H
2	757 cm^{-1}	H–O
3	729 cm^{-1}	H–O
4	718 cm^{-1}	H–O
5	696 cm^{-1}	H–O
6	678 cm^{-1}	H–O
7	621 cm^{-1}	H–O
8	469 cm^{-1}	H–O
9	451 cm^{-1}	O–H
10	401 cm^{-1}	O–H
11	375 cm^{-1}	O–H
12	346 cm^{-1}	O–H–Y
13	325 cm^{-1}	O–Y–H
14	306 cm–1	Y–H–O
15	296 cm^{-1}	O–H
16	279 cm^{-1}	O–Y–H
17	264 cm^{-1}	O–Y–H
18	259 cm^{-1}	O–H
19	218 cm^{-1}	O–H
20	198 cm^{-1}	O–H
21	163 cm^{-1}	Y–O–H
22	145 cm^{-1}	Y–O–H
23	143 cm^{-1}	Y–O

References

1. Mouhat, F.; and Coudert, F.-X. Necessary and sufficient elastic stability conditions in various crystal systems. *Phys. Rev. B,* **2014**, *90*, 224104.
2. Hill, R. The elastic behavior of a crystalline aggregate. *Proc. Phys. Soc. Sect. A* **1952**, *65*, 349.
3. Ranganathan, S.I.; Ostoja-Starzewski, M. Universal elastic anisotropy index. *Phys. Rev. Lett.* **2008**, *101*, 055504.
4. Kube, C. M. Elastic anisotropy of crystals. *AIP Adv.* **2016**, 6, 095209.
5. Voigt, W. *Lehrbuch der Kristallphysik (mit Ausschluss der Kristalloptik)*; Vieweg/Teubner Verlag: Braunschweig, Germany, 1966.
6. Reuss, A. Berechnung der fließgrenze von mischkristallen auf grund der plastizit¨atsbedingung f¨ur einkristalle. *ZAMM-J. Appl. Math. Mech.* **1929**, *9*, 49–58.
7. Chen, X.-Q.; Niu, H.; Li, D.; Li, Y. Modeling hardness of polycrystalline materials and bulk metallic glasses. *Intermetallics* **2011**, *19*, 1275–1281.
8. Tian, Y.; Xu, B.; Zhao, Z. Microscopic theory of hardness and design of novel superhard crystals. *Int. J. Refract. Met. Hard Mater.* **2012**, *33*, 93–106.
9. Pettifor, D.G. Theoretical predictions of structure and related properties of intermetallics. *Mater. Sci. Technol*. **1992**, *8*, 345–349.
10. Anderson, O.L. A simplified method for calculating the debye temperature from elastic constants. *J. Phys. Chem. Solids* **1963**, *24*, 909–917.
11. Belomestnykh, V.N.; Tesleva, E.P. Interrelation between anharmonicity and lateral strain in quasi-isotropic polycrystalline solids. *Tech. Phys*. **2004**, *49*, 1098–1100.

© 2020 by the authors. Submitted for possible open access publication under the terms and conditions of the Creative Commons Attribution (CC BY) license (http://creativecommons.org/licenses/by/4.0/).
